# Integrating Docking, Dynamics, and Assays to Predict Antimicrobial Peptide Interactions with Mycolic Acid Membranes in *Mycobacterium tuberculosis*

**DOI:** 10.1021/acsmeasuresciau.5c00126

**Published:** 2025-10-14

**Authors:** Cesar Augusto Roque-Borda, Oswaldo Julio Ramirez Delgado, Laura Maria Duran Gleriani Primo, Emma Dyhr, Ingvill Pedersen Sæbø, Emily Helgesen, James Booth, Henrik Franzyk, Paul R. Hansen, Hernan Morales-Navarrete, Beatriz G. de la Torre, Fernando Albericio, João Perdigão, Fernando Rogério Pavan

**Affiliations:** † Department of Biological Sciences, School of Pharmaceutical Sciences, 28108 Universidade Estadual Paulista (UNESP), 14800901 Araraquara, Brazil; ‡ iMed.ULisboa−Institute for Medicines Research, Faculty of Pharmacy, 37809 University of Lisbon, 1649004 Lisbon, Portugal; § Faculty of Health and Medical Sciences, Department of Drug Design and Pharmacology, 4321 University of Copenhagen, 2100 Copenhagen, Denmark; ∥ Department of Microbiology, 6305 Oslo University Hospital and the University of Oslo, Rikshospitalet, 0373 Oslo, Norway; ⊥ Department of Clinical and Molecular Medicine, Norwegian University of Science and Technology, and Clinic of Laboratory Medicine, St. Olavs Hospital, 7491 Trondheim, Norway; # Bio-Cheminformatics Research Group, Universidad de Las Américas, 170504 Quito, Ecuador; ¶ School of Laboratory Medicine and Medical Sciences, College of Health Sciences, 56394 University of KwaZulu-Natal, 4041 Durban, South Africa; ∇ Peptide Science Laboratory, School of Chemistry and Physics, University of KwaZulu-Natal, 4001 Durban, South Africa; ○ Department of Organic Chemistry, University of Barcelona, 08028 Barcelona, Spain

**Keywords:** antimicrobial peptides, *Mycobacterium tuberculosis*, multidrug resistance, membrane-targeting agents, molecular dynamics simulations, structure–activity relationship

## Abstract

The global burden of multidrug-resistant tuberculosis (MDR-TB) underscores the urgent need for novel therapeutics with distinct mechanisms of action. Here, we report a comparative evaluation of four antimicrobial peptides (AMPs) derived from the amphibian peptide B1CTcu5, integrating experimental validation with molecular modeling to elucidate structure–activity relationships. Among them, W-B1CTcu5, featuring a single N-terminal tryptophan substitution, exhibited the most potent antimycobacterial activity (MIC = 3.2 μg/mL) against *Mycobacterium tuberculosis* (MTB) combined with high structural stability, persistent membrane interaction, and multitarget affinity against key MTB proteins, including the porin MspA, the transporter CpnT, and the cell wall enzyme Ag85B. In contrast, analogs with reduced hydrophobic anchoring or dynamic instability demonstrated diminished efficacy despite partial membrane insertion or surface affinity. Molecular dynamics simulations revealed that peptides with low root-mean-square deviation and minimal residue fluctuation retained compact, α-helical conformations and maintained productive bilayer engagement, which are traits correlated with antimicrobial performance. However, the hemolytic properties of W-B1CTcu5 highlight a therapeutic trade-off between potency and host toxicity. Together, these findings emphasize the predictive power of dynamic structural descriptors in AMP design, and identify W-B1CTcu5 as a promising, yet optimization-requiring, scaffold for future design of anti-TB AMPs.

## Graphical abstract

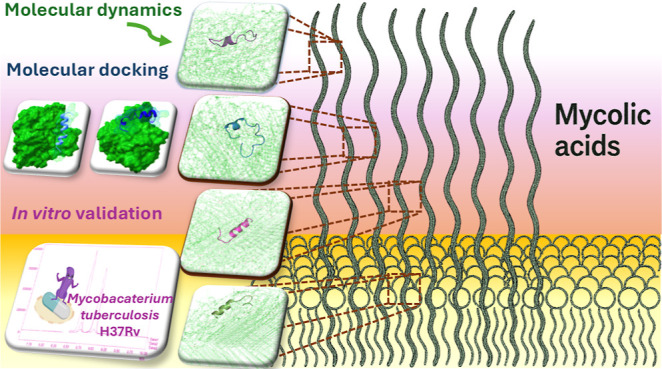

## 1 Introduction

Tuberculosis (TB), caused by *Mycobacterium tuberculosis* (MTB), continues to pose a significant global health burden, with over 10 million new cases and 1.3 million deaths annually.^1,2^ Despite existing curative anti-TB chemotherapy, the rise of multidrug-resistant (MDR) and extensively drug-resistant (XDR) MTB strains appears critically to undermine treatment efficacy. In particular, rifampicin-resistant TB represents a clinical and epidemiological inflection point, since it frequently indicates resistance to additional first- and second-line agents.^3^ Patients infected with such strains face prolonged treatment courses, increased toxicity, elevated costs, and substantially reduced cure rates.^4^ Compounding this crisis, there is a stagnation in TB drug discovery, with few candidates exhibiting mechanisms of action that circumvent existing resistance pathways, and hence mechanistically distinct antimicrobials are urgently needed.

The mycobacterial cell envelope constitutes a formidable barrier to antimicrobial agents due to its unique tripartite architecture, composed of an inner membrane, a complex arabinogalactan-peptidoglycan matrix, and an outer membrane rich in mycolic acids, which are long-chain fatty acids that account for 30–40% of the outer membrane’s composition and confer extreme hydrophobicity and rigidity to the barrier.^5–7^ These structural lipids not only reduce permeability to polar compounds but also play critical roles in virulence and immune modulation.^8,9^ While prior computational studies have explored the conformational and permeability properties of mycolic acids,^10^ the dynamic interactions between antimicrobial peptides (AMPs) and mycolic acid-enriched membranes remain unexplored.

AMPs have re-emerged as promising candidates in the postantibiotic era, especially against persistent intracellular pathogens such as MTB.^11^ Depending on their sequence and structural conformation, AMPs may act by disrupting bacterial membranes, binding to essential proteins, and thereby avoiding traditional resistance mechanisms, which are features that make certain AMPs promising candidates for therapeutic development.^12,13^ Due to their endogenous origin and capacity to modulate host immunity, AMPs have also been proposed as adjunct therapies. However, their clinical translation remains constrained by issues such as low selectivity, proteolytic instability, and, sometimes, cytotoxicity.^14^ These challenges are particularly pronounced in the context of TB, where the complex cell wall and intracellular localization both represent formidable limitations for activity.^15^

B1CTcu5, a 21-residue AMP derived from the skin of *Clinotarsus curtipes*, exhibits modest antimycobacterial activity, but suffers from pronounced hemolysis, limiting its therapeutic applicability.^16,17^ Nevertheless, its defined amphipathic structure offers a valuable scaffold for rational modification. Although prior studies suggest that tuning hydrophobicity, charge, or aromatic residues can enhance activity, the underlying structural determinants driving its interaction with MTB membranes and intracellular targets remain inadequately characterized.^16,18^

Here, we report on the rational redesign of B1CTcu5 into three novel analogs engineered for enhanced efficacy and selectivity against MTB. To dissect their mechanism of action in the context of the unique MTB outer membrane, they were tested on a customized α-mycolic acid-enriched bilayer model developed to mimic the biophysical barrier posed by the pathogen’s cell envelope, which allowed high resolution of AMP–membrane interactions under conditions reflecting MTB physiology. Hence, we evaluated W-B1CTcu5 (Trp-modified), CR2111 (amphipathically balanced), and CR2106 (conformationally dynamic) through in vitro susceptibility assays and advanced MD simulations. Further, their structural engagement with MTB membrane proteins (MspA, CpnT, and Ag85B) was studied in order to map the functional landscape of AMP–bacterium interaction. This integrative approach defines how peptide structure governs membrane behavior, conformational persistence, and target selectivity, which are critical features for advancing AMP-based therapies against MDR-TB.

## 2 Material and Methods

### 2.1 Chemical Reagents

Middlebrook 7H9 broth was purchased from Kasvi (Paraná, Brazil). Catalase was obtained from Thermo Fisher Scientific Inc. (MA, USA), and bovine serum albumin (BSA) was provided by Interlab Confiança (São Paulo, Brazil). Cell culture reagents including Roswell Park Memorial Institute medium (RPMI 1640, Gibco, lot number 2023270), Dulbecco’s Modified Eagle’s Medium (DMEM), fetal bovine serum (FBS), penicillin, and streptomycin were obtained from Gibco-Invitrogen (Thermo Fisher Scientific, USA). Analytical-grade reagents such as dextrose, *N*,*N*-dimethylformamide (DMF), dichloromethane (DCM), *N*,*N*′-diisopropylcarbodiimide (DIC), and 1-hydroxybenzotriazole hydrate (HOBt) were acquired from Sigma-Aldrich (MO, USA). Fmoc-protected amino acids, and all other SPPS reagents, trifluoroacetic acid (TFA), and acetonitrile (ACN) were of analytical grade and sourced from Sigma-Aldrich Co. (MO, USA).

### 2.2 Peptide Synthesis

The peptides W-B1CTcu5 (H-WLIAGLAANFLPQILCKIARKC-NH_2_), CR2111 (H-LIAGLAANFLPQILSKIARKA-NH_2_), and CR2106 (H-WLIAGLAANFLPQILSKARKS-NH_2_) were synthesized via manual Fmoc-based solid-phase peptide synthesis (SPPS) on a Rink Amide MBHA resin (0.5 mmol scale) according to Roque-Borda et al.^19^ The resin was preswelled for 15 min in a 1:1 solution of DMF–DCM prior to SPPS. Fmoc deprotection was carried out with 20% 4-methylpiperidine in DMF, and each Fmoc-protected amino acid building block (Fmoc-AA-OH) was coupled manually by using a 3-fold molar excess, which was activated with diisopropylcarbodiimide (DIC)/1-hydroxybenzotriazole (HOBt) in DMF and then coupled for 2 h. Upon assembly of the sequence and final Fmoc removal, the peptide was cleaved from the resin by treatment with a cleavage cocktail consisting of TFA/H_2_O/TIS (95:2.5:2.5, v/v/v) for 2 h at room temperature. The crude peptides were precipitated with cold diethyl ether and centrifuged (3000*g*, 5 min, repeated twice) followed by extraction with 30% acetic acid.

Analytical and preparative RP-HPLC were used to confirm the purity and identity of the peptides. Crude peptide analysis was carried out by using a Zorbax Eclipse XDB-C18 column (9.4 × 250 mm, 5 μm particle size) with a linear gradient elution from 5% to 95% solvent B over 30 min at a flow rate of 0.6 mL/min, monitoring absorbance at 220 and 280 nm. Solvent A consisted of 0.045% TFA in H_2_O, and Solvent B was 0.036% TFA in ACN at a constant flow rate of 2.0 mL/min. Purified peptides Were analyzed by electrospray ionization mass spectrometry (ESI-MS, positive mode) using a Bruker Amazon Ion Trap instrument. Additional chromatographic analysis was performed by using a Shimadzu LC-10A/C-47A HPLC system with a Waters Symmetry C18 column (2.1 × 150 mm, 15 μm) at room temperature (25 °C), to confirm molecular mass and peptide integrity. Peptide fractions were monitored at 220 nm, collected, and lyophilized.

Peptides B1CTcu5 (H-LIAGLAANFLPQILCKIARKC-NH_2_) was synthesized via Fmoc-based SPPS using automated platforms (CEM Liberty Blue microwave synthesizer or a Gyros Protein Technologies PurePep Chorus synthesizer with thermal heating). Synthesis was performed on H-Rink-amide resin (Matrix Innovation; loading 0.50 mmol/g, 0.1 mmol scale), using the same standard side-chain protecting groups then in the previous synthesis. Amino acid couplings were carried out in DMF with DIC (0.5 M in DMF) and OxymaPure (0.5 M in DMF) as activators. For the Liberty Blue system, 5.0 equiv of Fmoc-protected amino acid building blocks were used, while 3.0 equiv were used on the Gyros synthesizer. Fmoc-Arg­(Pbf)-OH was triple-coupled under peptide-specific conditions: either at 75 °C for 10 min (e.g., for W-B1CTcu5), or using a room temperature step (30 min) followed by brief microwave heating (2 min at 75 °C). All other residues were double-coupled at 75 °C for 10 min, except Fmoc-Ile-OH that was triple-coupled. Fmoc removal was performed by using 20% piperidine in DMF (2 × 3 min at 75 °C). Peptides synthesized via automated SPPS were cleaved from the resin and deprotected by using the same protocol as described above for manual SPPS. Crude products were subsequently concentrated under reduced pressure, coevaporated with toluene, and purified by preparative HPLC, which was performed on a Phenomenex Luna C18(2) column (250 × 21.2 mm; particle size: 5 μm; pore size 100 Å) on a Shimadzu Prominence system, eluting with H_2_O–MeCN gradients with 0.1% trifluoroacetic acid (TFA) added to eluents A (5:95 MeCN–H_2_O + 0.1% TFA) and B (95:5 MeCN–H_2_O + 0.1% TFA) with UV detection at λ = 220 nm at room temperature (25 °C). The purity of each peptide was assessed by analytical HPLC using a Phenomenex Luna C18 HST column. The analysis employed the same solvent system as the preparative HPLC, with a linear gradient elution from 0% to 60% solvent B over 15 min, at a flow rate of 0.5 mL/min.

### 2.3 Antimicrobial Activity

Antimycobacterial activity was evaluated by using the reference strain MTB H37Rv (ATCC 27294). The strain was cultured in Middlebrook 7H9 broth supplemented with oleic acid, bovine serum albumin fraction V, dextrose, and catalase. Cultures were maintained under agitation at 200 rpm and 37 °C for up to 3 weeks until reaching log-phase growth. The MIC of each peptide was determined by the resazurin microtiter assay (REMA) in 96-well plates, following Clinical and Laboratory Standards Institute (CLSI, 2018) guidelines.^20^ After 1 month of incubation, MTB H37Rv cultures were adjusted to a turbidity equivalent to 1.0 McFarland standard (∼3 × 10^8^ cfu/mL). Peptides were tested at serial 2-fold dilutions ranging from 250 to 0.098 μg/mL in 7H9-supplemented medium. Rifampicin and isoniazid were used as drug controls in concentrations ranging from 25 to 0.098 μg/mL. Plates were incubated for 7 days at 37 °C in a 5% CO_2_ atmosphere. After incubation, 30 μL of 0.01% resazurin solution was added to each well, and fluorescence was measured after 24 h by using a Synergy H1 microplate reader (BioTek, USA) at excitation/emission wavelengths of 530/590 nm. All experiments were performed in biological triplicates. As part of the antimicrobial profiling, the peptides were also tested against *Escherichia coli* ATCC 25922 and *Staphylococcus aureus* ATCC 29213 under standard REMA assay conditions.

#### 2.3.1 Cytotoxicity Assays

Cytotoxic activity was evaluated in J774A.1 murine macrophages (ATCC TIB-67, 10 passages) and MRC-5 human lung fibroblasts (ATCC CCL-171, 12 passages) by using the resazurin-based AlamarBlue viability assay.^21^ Cells were cultured in RPMI 1640 (for J774A.1) or DMEM (for MRC-5) media supplemented with 10% (v/v) fetal bovine serum, gentamicin (75 μg/mL), and amphotericin B (3 μg/mL), under standard incubation conditions (37 °C, 5% CO_2_). For the assay, cells were seeded into 96-well plates at a density of 2.5 × 10^5^ cells/mL (RPMI) or 1 × 10^6^ cells/mL (DMEM) and incubated for 24 h. Peptide samples were diluted to 3.2% in phosphate-buffered saline (PBS, pH 7.4, 1:9 v/v) and applied to the cells for 24 h. After treatment, cell viability was quantified by adding AlamarBlue reagent and reading fluorescence at 530/590 nm determined by REMA (IC_50_ values) in biological triplicates.

Hemolytic activity was determined by using freshly collected human blood. Peripheral blood was drawn from a healthy volunteer into 4 mL lithium heparin tubes (Greiner Bio-One) using a 23 G butterfly needle attached to a 19 cm luer adapter. The blood was centrifuged at 1700*g* for 5 min, and the plasma was removed. Erythrocytes were washed three times with PBS at pH 7.0 until the supernatant was clear. The washed pellet was diluted 1:100 in PBS to prepare a 1% erythrocyte suspension. In a 96-well polypropylene PCR plate, 50 μL of peptide solution, PBS (negative control), or 10% Triton X-100 (positive control) were mixed with 50 μL of the erythrocyte suspension. After incubation at 37 °C for 60 min, samples were centrifuged again at 1700*g* for 5 min. Then, 50 μL of the supernatant was transferred to a flat-bottom 96-well plate (Anicrin), and the absorbance at 405 nm was measured by using a Victor Nivo microplate reader (PerkinElmer). The percentage of hemolysis was calculated by using the formula

1 $$\%\;hemolysis=\frac{{OD}_{test}-{OD}_{neg}}{{OD}_{pos}-{OD}_{neg}}$$

where OD_test_ is the absorbance of the sample treated with the peptide, OD_neg_ is the absorbance of the negative control (PBS), and OD_pos_ is the absorbance of the positive control (10% Triton X-100). Hemolysis was expressed as a percentage relative to complete lysis induced by the positive control. Assays were performed for B1CTcu5 and W-B1CTcu5 only, as these peptides presented higher in vitro antimycobacterial activity and were prioritized for structural characterization. CR2111 and CR2106 were not evaluated in this assay phase due to their moderate or low bioactivity.

### 2.4 Molecular Docking

#### 2.4.1 Modeling of Mycolic Acids and AMPs

The structure of α-mycolic acid was generated from its SMILES representation retrieved from the ChEBI database (CHEBI ID: 59235; https://www.ebi.ac.uk/chebi/CHEBI:59235). Molecular topology was prepared using the Automated Topology Builder to generate parameters compatible with the GROMOS force field, which was selected for its validated performance in simulating drug-mycolic acid interactions under pressure.^10^ A lipid bilayer was then constructed by using MEMGEN, consisting of 100 α-mycolic acid molecules per leaflet, solvated with 50 SPC water molecules per lipid, and configured with an area per lipid of 58 Å^2^, following previously validated parameters.^22^ Four AMPs characterized for their anti-MTB activity (see Sections Peptide Synthesis and Antimicrobial Assays) were selected for structural modeling. Their 3D conformations were predicted by using AlphaFold3 and evaluated based on pLDDT confidence scores and Ramachandran plots to ensure structural reliability for downstream simulations (Figure S1). Theoretical physicochemical parameters (net charge, instability index, aliphatic index) were calculated using the Expasy ProtParam server and https://www.pepcalc.com/.

#### 2.4.2 Outer Membrane Protein Retrieval, Modeling, and Molecular Docking

Relevant outer membrane proteins were identified through a systematic literature review and protein database mining (Table S1). The proteins included the PE/PPE protein complex from MTB (PDB: 2G38), Porin MspA (PDB: 1UUN), Rv1698 (AlphaFold DB: P9WJ83; mycobacterial copper transport protein B), and CpnT (AlphaFold DB: O05442). CpnT exhibits a dual function in nutrient uptake and induction of host cell death: its N-terminal domain (NTD) forms an outer membrane channel that facilitates nutrient transport, while the secreted C-terminal toxic domain (TNT) acts as a glycohydrolase that hydrolyzes the essential coenzyme NAD^+^ in the cytosol of infected macrophages, thereby causing necrotic host cell death. Both domains are essential for survival, replication, and cytotoxicity of MTB within macrophages. When available, experimentally resolved 3D structures were obtained from the Protein Data Bank; otherwise, structures were predicted de novo using AlphaFold3 based on primary amino acid sequences (Table S1). Molecular docking was performed with LightDock, which applies a Glowworm Swarm Optimization algorithm for conformational sampling. To incorporate protein flexibility, backbone mobility was modeled using the Anisotropic Network Model. Docked complexes were refined through 100 steps of energy minimization with the Amber99SB force field. The top five docking poses for each peptide–protein pair were selected based on combined energetic and geometric criteria. Binding affinities were then estimated with the contact-based scoring function in PRODIGY,^23^ and receptors showing binding free energy values greater than −9 kcal/mol were prioritized for in-depth interaction analysis.

Among the selected proteins, MspA was prioritized as the prototypical porin mediating hydrophilic solute diffusion across the outer membrane, while CpnT was included due to its dual role in nutrient uptake and induction of host cell death. Ag85B was considered for its essential enzymatic function in mycolic acid transfer reactions, directly contributing to cell wall biosynthesis and envelope integrity. In contrast, PE/PPE proteins and Rv1698 were retrieved for completeness as surface-exposed or membrane-associated proteins identified in database searches, but the comparative binding analyses focused primarily on MspA, CpnT, and Ag85B, given their well-established roles in MTB physiology and virulence.

#### 2.4.3 Molecular Dynamics Simulations

AMPs were initially positioned at coordinates (3.80790, 3.80790, 14.00000) within a simulation box of dimensions 7.61580 × 7.61580 × 15.34000 nm^3^. All simulations were conducted in GROMACS 2024 using the GROMOS 54A7 force field. The simulation protocol consisted: (i) energy minimization with 20,000 steps via the steepest descent algorithm and a convergence criterion of 1000 kJ/mol/nm; (ii) a 10 ns NPT equilibration phase with positional restraints applied to peptides to allow system relaxation and solvent adaptation;^24^ and (iii) a 100 ns production run under physiological conditions (310 K, 1 bar). Long-range electrostatic interactions were treated with Particle Mesh Ewald (PME) method, with both Coulomb and van der Waals cutoffs set at 1.2 nm for all phases.

##### 2.4.3.1 Structural Stability

Root mean square deviation (RMSD) and root-mean-square fluctuation (RMSF) were calculated for each peptide.

##### 2.4.3.2 Center-of-Mass (COM) Dynamics

Time-dependent COM distances between peptides and the mycolic acid bilayer were calculated by using Newtonian Equations of motion [Disp-formula eq2] and 3

2 $$r_{i}=\frac{p_{i}}{m_{i}}$$

3 $$p_{i}=-Vr_{i}\times V=f_{i}$$

where *r*_*i*_ and *p*_*i*_ represent the position and linear momentum of particle *i* mass (*m*_*i*_), *V* is the system’s potential energy and *f*_*i*_ the net force. For selected atom groups, the COM position was determined via eq 4

4 $$R\left(t\right)=\frac{\sum_{i=1}^{N}m_{i}r_{i}\left(t\right)}{\sum_{i=1}^{N}m_{i}}$$

where *N* is the number of atoms. Absolute COM distance and relative displacement eqs 5 and 6

5 d(t)=|R(t)|

6 Δd(t)=|R(t)−R(0)|

All metrics of COM were computed using the gmx distance module in GROMACS. This analytical framework enabled us to establish quantitative correlations between atomic-scale dynamics (eqs 2 and 3) and mesoscopic interactions (eqs 4–6), providing mechanistic insight into AMP-membrane binding behavior.^25^

## 3 Results and Discussions

### 3.1 Antimycobacterial Efficacy and Host-Cell Selectivity

To improve the chemical stability and interpretability of biological assays involving B1CTcu5, two new analogs—CR2106 and CR2111—were designed based on targeted residue substitutions. The native peptide contains two cysteines that are susceptible to oxidation under ambient and physiological conditions, posing a challenge for applications that require prolonged incubation, such as antimycobacterial assays. In particular, standard MTB inhibition protocols extend over at least 7 days, during which oxidation and disulfide bridge formation may occur spontaneously. This can lead to unwanted peptide cyclization, altered activity profiles, and batch-to-batch variability, ultimately compromising both reproducibility and biological relevance. To mitigate these issues, CR2106 was generated by replacing the cysteines with serines, preserving the polarity and side-chain volume while eliminating redox sensitivity. This analog retained the *N*-terminal tryptophan introduced in W-B1CTcu5, previously shown to enhance antimycobacterial activity, likely by promoting membrane interaction through its aromatic character. However, to maintain peptide length and avoid excessive *N*-terminal hydrophobicity, the adjacent isoleucine was removed. This adjustment allowed preservation of the amphipathic profile while minimizing aggregation potential during extended incubations.

In parallel, CR2111 was designed to evaluate whether more conservative modifications could improve peptide behavior without altering the N-terminal sequence. Here, cysteines were substituted by alanines, a minimal side-chain change frequently used to probe structural tolerance, while lysines were replaced by arginines to test the impact of guanidinium groups on charge distribution and target interaction. Unlike CR2106, this analog did not incorporate the N-terminal tryptophan, allowing a clearer dissection of how localized aromaticity versus backbone composition contributes to bioactivity. These two design strategies—stabilization through amphipathic adjustment in CR2106 and conservative structural simplification in CR2111—provided complementary insights into the sequence–activity relationships governing this peptide scaffold.

In this study, all four tested peptides, derived from a common amphipathic scaffold, demonstrated inhibitory activity against MTB H37Rv (Table 1), yet the degree of efficacy varied substantially according to specific residue modifications. The N-terminal insertion of Trp, a hydrophobic residue, enhanced antimycobacterial activity when introduced into the native sequence, consistent with its known role in promoting membrane interaction. However, in a second analog where Trp was retained but additional modifications were made—including removal of an adjacent Ile and substitution of both Cys residues by Ser—the enhanced activity was not preserved. This divergence suggests that the effects of Trp insertion are context-dependent, and that multiple structural elements may interact in nonlinear ways to influence potency, particularly when balancing hydrophobicity, amphipathicity, and sequence length.^26^

**Table 1:**
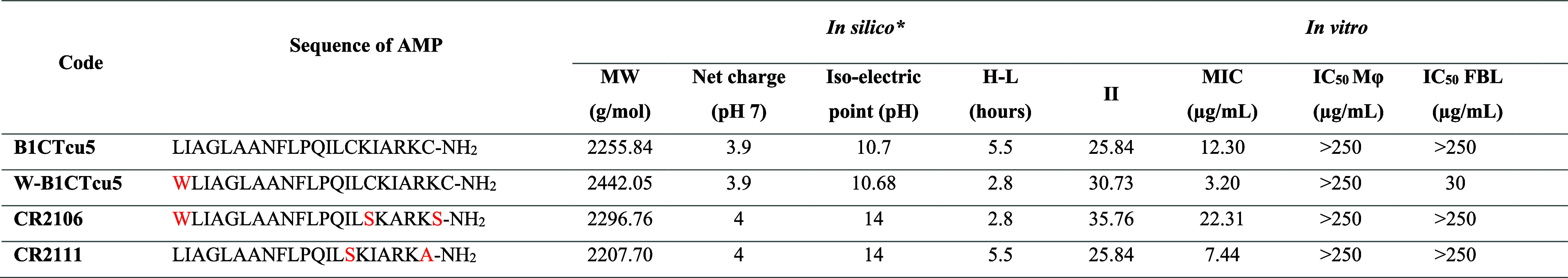
Antimycobacterial Activity and Cytotoxicity Profiles of B1CTcu5-Derived AMPs

a In silico studies was predicted using https://pepcalc.com/ and https://web.expasy.org/protparam. MW: Molecular weight. H-L: Estimated half-life in mammalian reticulocytes. II: Instability index. MIC: Minimal inhibitory concentration. IC_50_: Half-maximal Inhibitory Concentration. Mφ: Macrophages. FBL: Fibroblasts.

Most peptides demonstrated minimal activity against *E. coli* and *S. aureus*, supporting a narrow–spectrum profile (Table 2). However, CR2111 was a notable exception, with a MIC of 11 μM against *E. coli* and 8 μM against *S. aureus*. These data suggest that, unlike its Trp-modified counterparts, CR2111 may engage more conserved bacterial surface features, warranting further evaluation of its spectrum. This sharply contrasts with many conventional AMPs and instead suggests selective recognition of MTB-specific features, e.g., its mycolic acid–rich outer membrane and unique porins like MspA and CpnT.^27^ Such specificity is increasingly valued in antimicrobial development, as it reduces off-target microbiota disruption and mitigates the emergence of resistance driven by broad-spectrum selection pressure.^28^

**Table 2:** Antimicrobial Activity in Other Bacteria

	E. coli MIC		S. aureus MIC	
code	(μM)	(μg/mL)	(μM)	(μg/mL)
B1CTcu5	>125	>281.98	>125	>281.98
W-B1CTcu5	>250	>610.51	>250	>610.51
CR2106	>32	>73.50	16	36.75
CR2111	11	24.28	8	17.66

Cytotoxicity assays in J774A.1 macrophages and MRC-5 fibroblasts revealed a divergent profile among the analogs. All peptides were well tolerated by macrophages, yet one analog, W-B1CTcu5, exhibited significant toxicity in fibroblasts, in line with its increased amphipathicity and enhanced membrane affinity.^29^ This analog also exerted the most pronounced hemolysis at the high test concentration of 400 μg/mL, raising concerns about its systemic compatibility despite its superior antimycobacterial performance. In contrast, CR2111 and CR2106 maintained low cytotoxicity across both cell lines and were not hemolytic under the tested conditions, suggesting a more favorable safety margin.^30^ Although only a subset of analogs advanced to in-depth toxicological assessment, this reflects a strategic decision to focus on candidates with both high antimycobacterial potency and favorable preliminary safety profiles.^31^

While CR2106 and CR2111 displayed negligible erythrocyte lysis at 400 μg/mL (<5%), W-B1CTcu5 caused almost complete hemolysis (93.1%) under the same conditions, underscoring a critical safety concern. This disproportionate effect is consistent with the physicochemical shift introduced by the *N*-terminal Trp, which increases hydrophobic surface density and facilitates deeper bilayer insertion. Such insertion reduces the peptide’s ability to discriminate between anionic bacterial membranes and zwitterionic erythrocyte membranes, leading to loss of host selectivity. Hemolysis at this magnitude has historically been a major roadblock for the translational development of amphipathic AMPs, even when accompanied by favorable antimicrobial potency. In contrast, the low hemolytic activity of CR2106 and CR2111 indicates that subtle residue substitutions—Ser for Cys in CR2106 or Ala/Lys substitutions in CR2111—can attenuate host toxicity without abrogating antimycobacterial activity. These data collectively highlight how minor sequence adjustments modulate the delicate balance between potency and safety, and point to CR2106 and CR2111 as scaffolds with greater therapeutic promise.^32,33^

From a therapeutic standpoint, the narrow–spectrum profiles observed for all peptides support their potential as precision antimicrobials. The ability to selectively inhibit MTB without affecting representative Gram-positive or Gram-negative bacteria reinforces their relevance in a context where preserving host microbiota is prioritized. This narrow activity window, achieved through minimal residue substitutions, also maintains synthetic tractability, which is an often-overlooked advantage in early phase drug discovery.^34^ The hemolytic activity and chromatographic behavior of B1CTcu5 and its analogs were evaluated to determine the influence of structural modifications on peptide selectivity and physicochemical properties. As shown in Table 3, both CR2106 and CR2111 exhibited minimal hemolysis (<5%) compared to the parent peptides, while maintaining comparable retention times and elution profiles, suggesting improved biocompatibility without major alterations in hydrophobicity. The elevated cytotoxicity and hemolytic profile of W-B1CTcu5 exemplify a common pitfall in AMP development, where enhanced amphipathicity compromises host selectivity despite potent antimicrobial activity. This limitation underscores the challenge of balancing potency with safety, a trade-off that may only be addressed through rational sequence redesign or protective delivery strategies.^34^

**Table 3:** Hemolytic Activity and Chromatographic Properties of B1CTcu5 and Its Analogs^a^

code	hemolysis at 400 μg/mL (%)	retention time (*R*_t_)	% MeCN
B1CTcu5	75.20	14.45	57.00
W-B1CTcu5	93.10	13.99	63.80
CR2106	3.50	14.20	59.50
CR2111	3.00	14.40	56.80

a % MeCN: Percentage of acetonitrile.

These results collectively reinforce the principle that structural fine-tuning of AMPs must consider not only bacterial interactions, but also differential host membrane architecture.^34^ Here, CR2111 emerges as a promising lead, since it retains antimycobacterial efficacy comparable to the most potent analog while lacking severe toxicity, suggesting a more balanced activity profile for further development. W-B1CTcu5 exemplifies the potency–toxicity trade-off, underscoring the need for mitigation strategies such as PEGylation or targeted delivery to reduce off-target membrane disruption and improve hemocompatibility. While lipidation has been used to enhance antimicrobial activity, it may increase hemolytic potential if not properly balanced by increased net charge and a retained hydrophobicity.^35^

The observed differences in biological performance prompted further investigation into the molecular interactions of these peptides. Thus, we next performed high-resolution in silico analyses, including molecular docking and dynamics simulations in order to assess structural stability, membrane-binding behavior, and target engagement profiles of the peptide analogs. These approaches may provide mechanistic insight into how sequence-dependent biophysical traits translate into antimicrobial performance, and may contribute to the development of predictive tools for next-generation AMP design, pending broader validation (Figure S1).

### 3.2 Structural Characterization of Molecular Recognition by Porin MspA

Since a model of the outer membrane was subsequently analyzed to investigate the conformational behavior of the studied AMPs, the following outer membrane receptors (PE/PPE, Porin MspA, CpnT, Rv1698, and Ag85B) were selected after searches in the STRING, PFAM, and PDB databases. The most promising targets, according to their binding affinity values, were MspA, CpnT, and Ag85B (Table S1), whose interaction profiles are discussed below. To elucidate the mechanistic basis of the observed antimycobacterial selectivity, we examined the interaction of each AMP with MspA, a key hydrophilic channel in the outer membrane of MTB. Variations in the binding profiles provided insights into how sequence modifications may influence peptide–membrane interactions and, ultimately, biological performance. Among the analogs, W–B1CTcu5 showed the strongest predicted interaction with MspA (Δ*G* ≈ −9.6 kcal/mol).

To further substantiate the docking predictions, we compared the output from two complementary visualization tools, LigPlot+ and Discovery Studio, both of which generate 2D interaction maps but emphasize different features. Discovery Studio captured a broader diversity of noncovalent interactions, as illustrated in the figure legends,^36^ while LigPlot^+^ produced a more condensed representation, focusing on hydrogen bonds and hydrophobic contacts.^37^ This combined approach enabled a more accurate identification of critical residues and provided detailed information regarding the chemical nature of the interactions involved. In particular, GLU127B and GLN126A were consistently highlighted as key residues, forming stabilizing hydrogen bonds and hydrophobic interactions with the peptides.

It is noteworthy that Discovery Studio primarily depicted hydrogen-bonding interactions, whereas LigPlot^+^ was able to resolve both hydrogen bonds and hydrophobic contacts (Figures 1, 2 and Table S3). This complementary evidence reinforced the robustness of the docking analyses. Importantly, across both platforms, the N-terminal tryptophan residue (Trp1C) emerged as a pivotal determinant of molecular recognition, acting as a primary anchoring residue through a combination of hydrogen bonding, electrostatic contacts, and aromatic stabilization.

**Figure 1:**
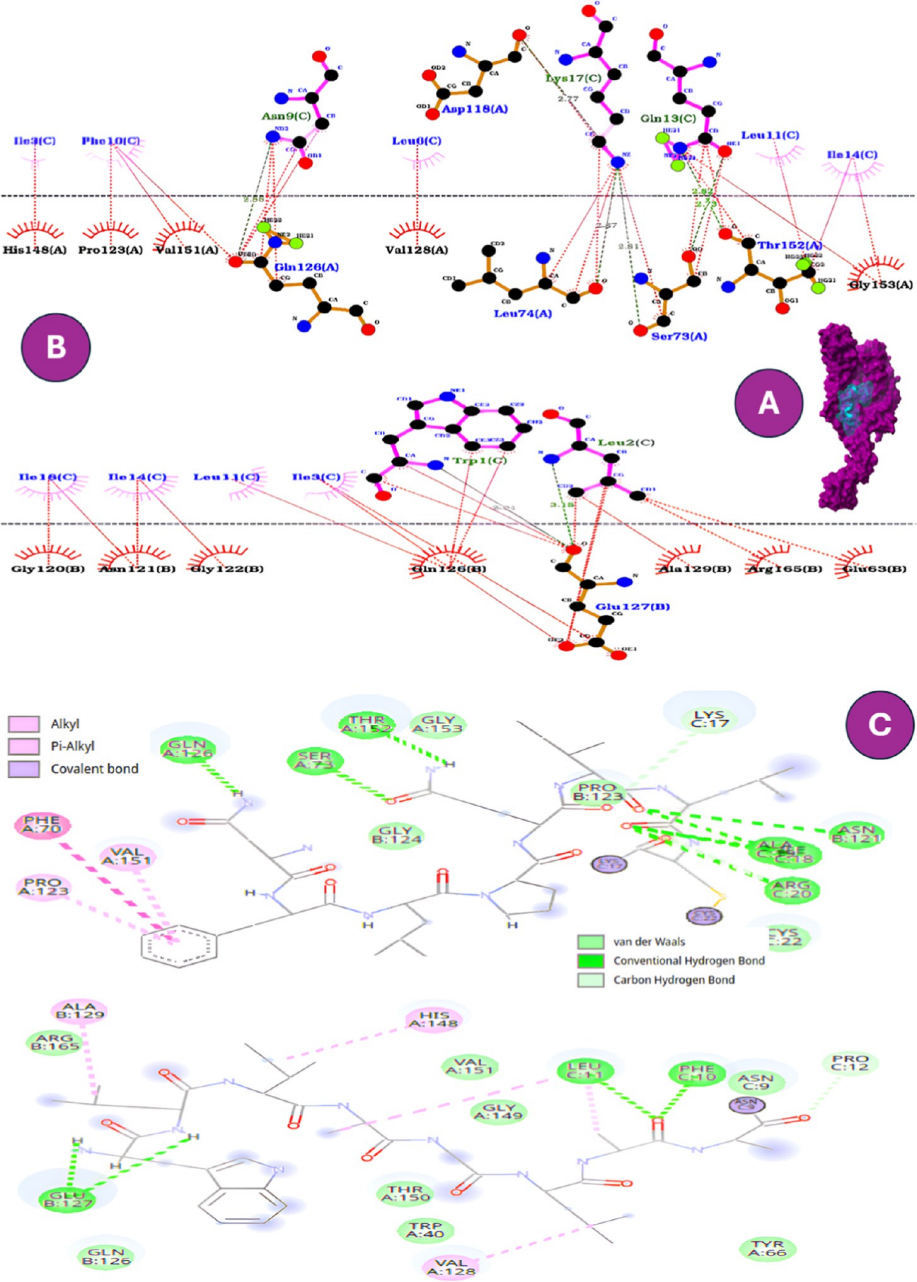
Comparative interaction profiles of W-B1CTcu5 docked into the mycobacterial porin MspA. (A) Surface representation of the peptide–protein complex. (B) Two-dimensional interactions generated in LigPlot^+^, highlighting hydrogen bonds (green dashed lines) and hydrophobic contacts (red arcs). (C) Two-dimensional interactions generated in Discovery Studio, showing an expanded spectrum of noncovalent interactions. In both visualization tools, the *N*-terminal tryptophan residue (Trp1C) emerges as a critical anchoring residue through contacts with GLU127B and GLN126A.

The interaction between the peptides and the MspA porin suggests a plausible mechanism of action involving channel obstruction and local destabilization of the outer membrane. The *N*-terminal tryptophan residue appears to play a pivotal role, promoting insertion through conventional hydrogen bonding. As shown in Figure 1, W-B1CTcu5 adopts a conformation favoring this type of bond, in contrast to other peptides (Figures 2B and 3C) that preferentially adopt α-helical conformations, which appear to correlate with superior anti-MTB activity.^34,35^ This observation highlights an inherent trade-off between potency and host compatibility.^38^ Among the analogs, CR2106 showed the highest binding affinity for MspA (−10.1 kcal/mol).^39,40^ This strong binding was stabilized by a salt bridge between GLU127B and the aromatic ring of Trp1C, in addition to hydrogen bonds with GLU63B (Figure 2). The anchoring effect of Trp was evident; however, its persistence may have been compromised by sequence modifications involving substitution of cysteines with serines (Table 1), potentially explaining the slight reduction in antimycobacterial efficacy despite the high affinity. Both LigPlot^+^ and Discovery Studio confirmed these key interactions, although Discovery Studio provided greater detail on the specific nature of noncovalent contacts.

**Figure 2:**
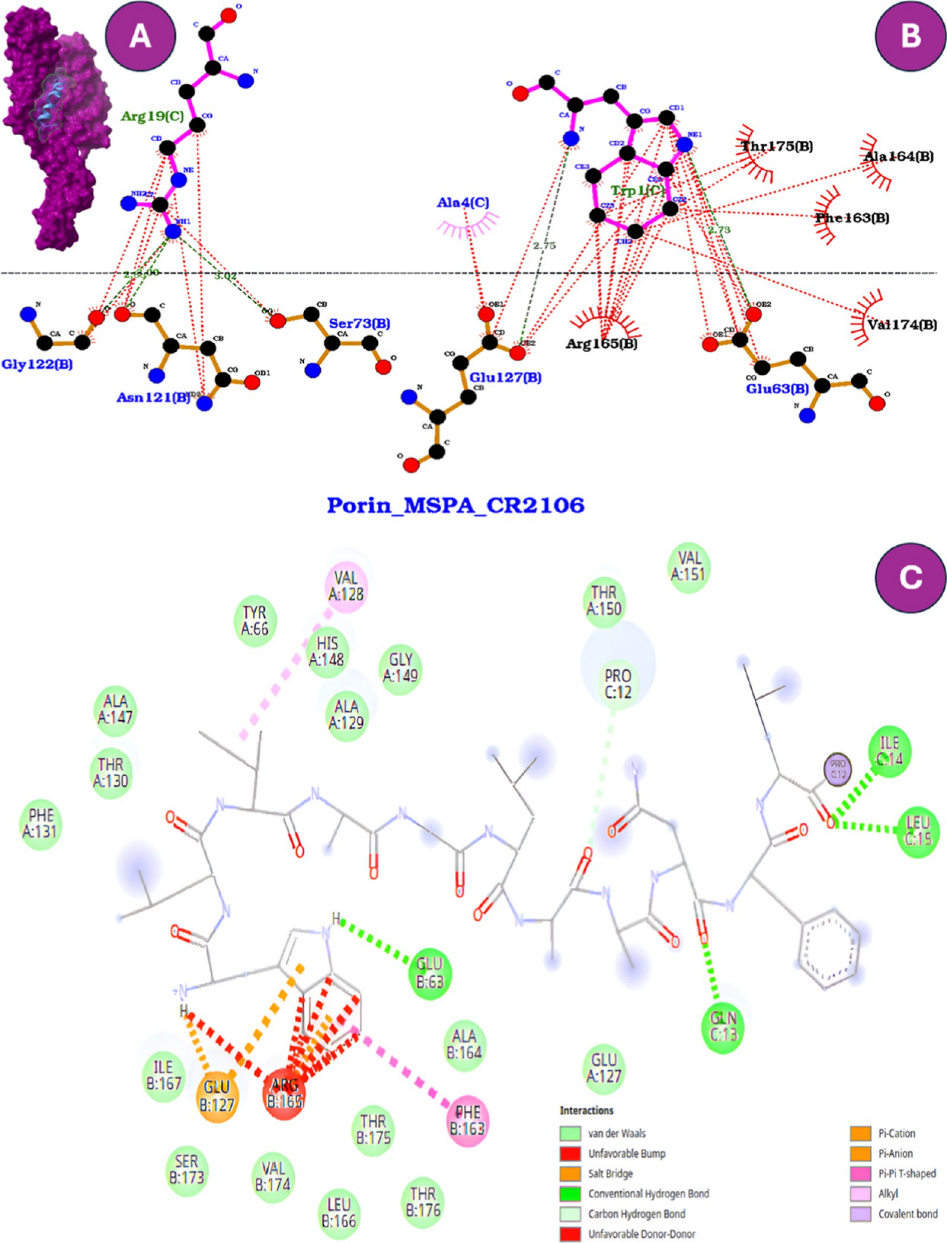
Comparative interaction profiles of CR2106 docked into the mycobacterial porin MspA. (A) Surface representation of the peptide–protein complex. (B) Two-dimensional interactions generated in LigPlot^+^, showing hydrogen bonds (green dashed lines) and hydrophobic contacts (red arcs), where Trp1C forms key interactions with GLU127B and GLU63B. (C) Two-dimensional interactions generated in Discovery Studio, highlighting an expanded interaction network, including salt bridges and additional noncovalent contacts. Both visualization tools consistently emphasize the anchoring role of Trp1C within the porin vestibule.

In contrast, B1CTcu5 and CR2111 displayed weaker binding affinities (−8.3 and −9.1 kcal/mol, respectively), with contacts limited to peripheral loops (Figure 3). Notably, both peptides lacked the stabilizing interaction with GLU127B that was present in W-B1CTcu5 and CR2106. This absence indicates that removal of the N-terminal Trp reduces the capacity to disrupt porin function or facilitate uptake, consistent with their lower antimicrobial activity.^41^ Taken together, these results suggest that N-terminal Trp insertion does not confer a universal gain-of-function, but rather enhances antimicrobial activity through stronger membrane and porin interactions, albeit at the expense of reduced selectivity.^34^ Importantly, while high-affinity interactions with MspA support a model of porin blockade, they cannot fully explain the activity differences observed among analogs, indicating that additional molecular targets are likely involved.^40^

**Figure 3:**
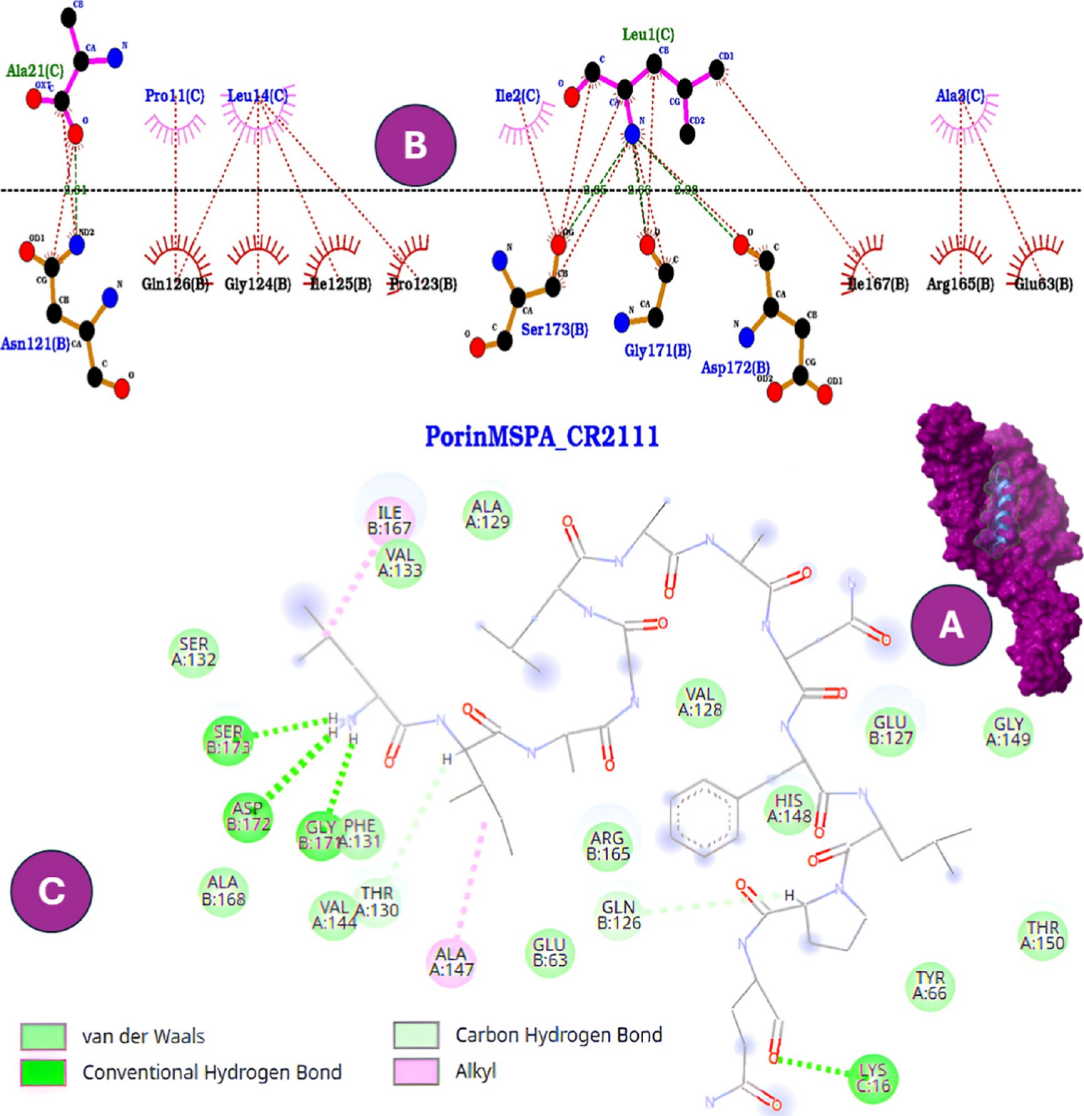
Comparative interaction profiles of CR2111 docked into the mycobacterial porin MspA. (A) Surface representation of the peptide–protein complex. (B) Two-dimensional interactions generated in LigPlot^+^, showing hydrogen bonds (green dashed lines) and hydrophobic contacts (red arcs). (C) Two-dimensional interactions generated in Discovery Studio, highlighting hydrogen bonds, alkyl interactions, and van der Waals contacts. Unlike W-B1CTcu5 and CR2106, CR2111 lacks the N-terminal tryptophan residue, preventing stable anchoring to GLU127B and resulting in weaker overall binding.

### 3.3 Hijacking CpnT to Impair Nutrient Entry and Lipid Barrier Adaptation in MTB

CpnT is an outer-membrane protein unique to MTB, where it is implicated in nutrient uptake and it is associated with cytotoxicity during intracellular infection. This dual function, along with its surface exposure, renders CpnT an attractive target for AMPs, as it may disrupt bacterial physiology beyond canonical membrane lysis.^42,43^ Structural studies have revealed that CpnT contributes to mycobacterial fitness by facilitating iron acquisition and participating in lipid remodeling, which both are essential for survival under host-imposed stress conditions.^44^ To explore whether this is relevant here, molecular docking was employed to evaluate peptide–CpnT interactions. W-B1CTcu5 exhibited the strongest affinity (−11.3 kcal/mol) through a network of well-defined polar, electrostatic, and hydrophobic contacts (Figure 4). In the N-terminal region of the peptide, ASP A:658 forms salt-bridge and π-anion interactions with the aromatic ring of tryptophan, constituting a key charged anchoring point. In addition, GLN A:660 establishes a conventional hydrogen bond, reinforcing polar fixation at this side. This interaction suggests an anchoring effect within the channel’s transport vestibule, plausibly interfering with iron translocation, thereby weakening bacterial adaptability under iron-limiting conditions.^43^

**Figure 4:**
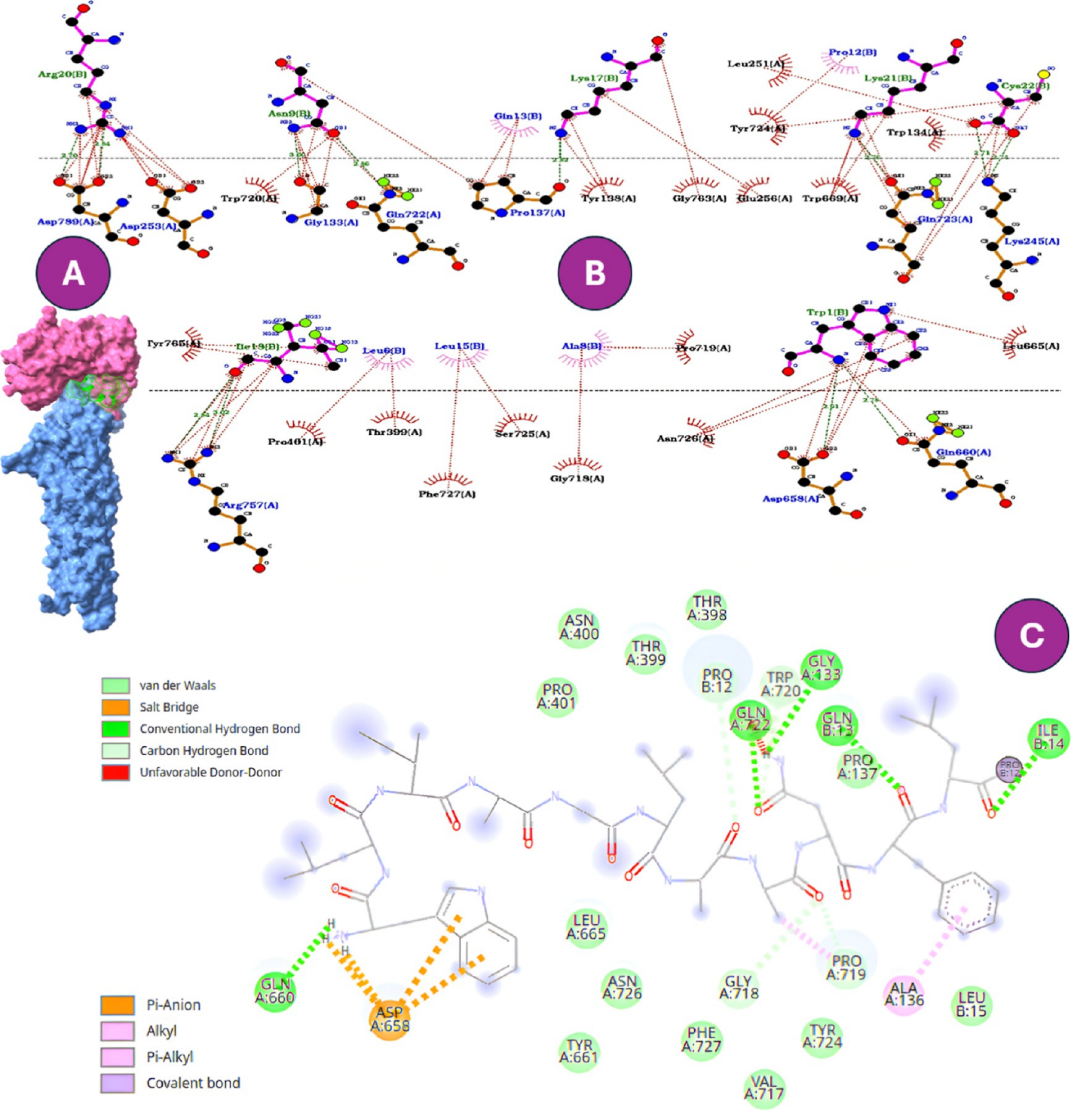
Predicted binding interactions of W-B1CTcu5 with CpnT, the outer-membrane nutrient transporter of MTB. (A) Surface representation of the docked complex. (B) Two-dimensional interaction map generated in LigPlot^+^, where the N-terminal tryptophan establishes salt-bridge and hydrogen-bond contacts with ASP658A and GLN660A. (C) Interaction map generated in Discovery Studio, highlighting additional stabilizing contacts with the same residues.

In contrast, B1CTcu5 showed weaker binding energy (−9.4 kcal/mol), with contact sites located primarily along peripheral residues (Figure 5). These more superficial interactions may reduce its capacity to inhibit nutrient uptake, aligning with its moderate antimicrobial performance.^45^ Notably, CR2111’s displayed even lower affinity for CpnT is consistent with its moderate antimycobacterial activity, reinforcing the relevance of interaction depth and multisite targeting in defining peptide efficacy and selectivity. Taken together, the possible interactions with both MspA and CpnT inferred from these modeling studies suggest a dual-action mechanism while MspA engagement may aid membrane traversal or ion flux perturbation, CpnT binding could disrupt nutrient homeostasis and exacerbate bacterial stress.^45,46^ Thus, these possible mechanisms for W-B1CTcu5 may contribute to its superior antimycobacterial activity.

**Figure 5:**
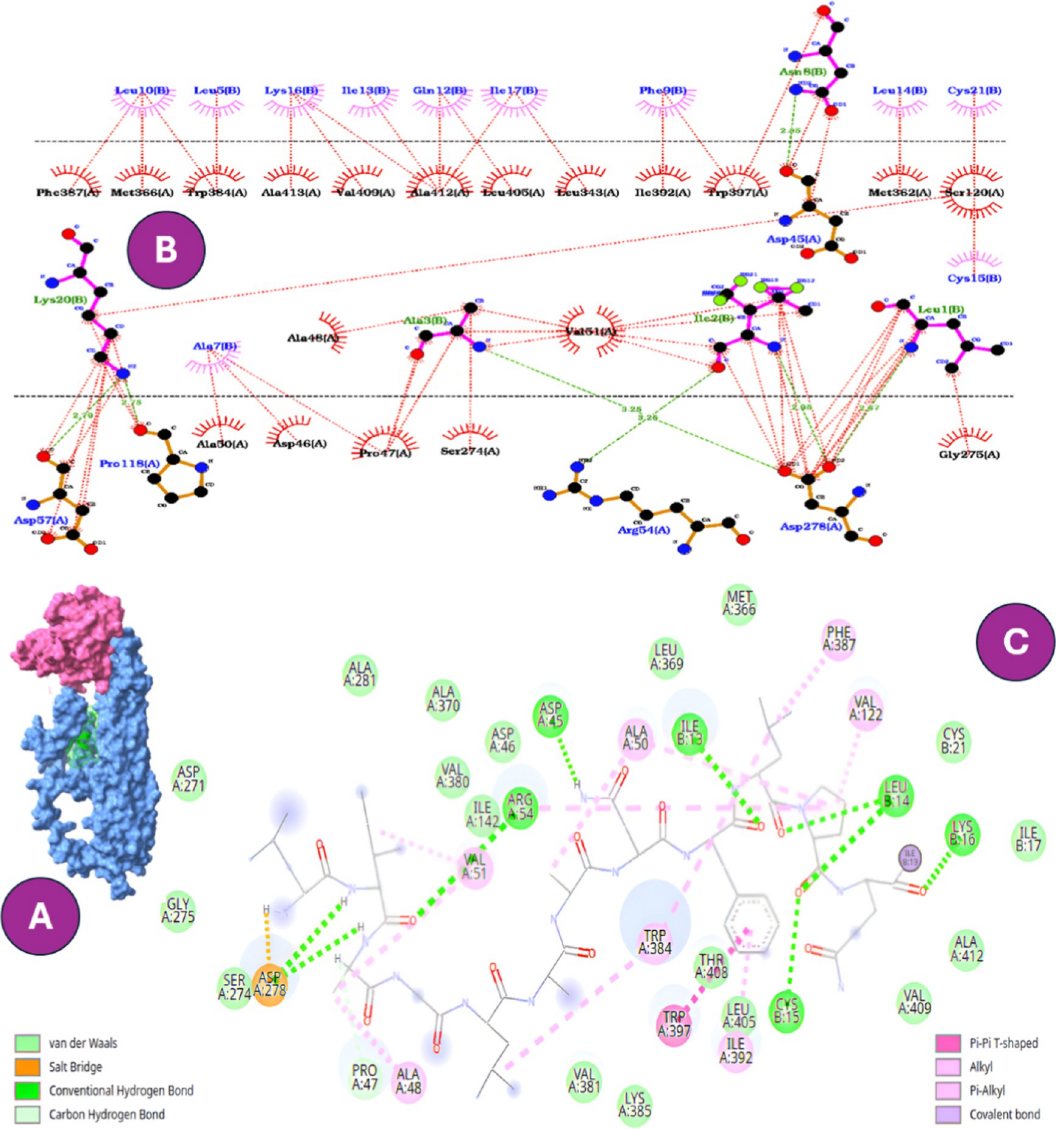
Predicted binding interactions of B1CTcu5 with CpnT, the outer-membrane nutrient transporter of MTB. (A) Surface representation of the docked complex. (B) Two-dimensional interaction map generated in LigPlot^+^, showing conserved polar interactions (ASP45A, ASP278A, ARG54A, LYS16B) through hydrogen bonds and salt bridges, and hydrophobic contacts involving ILE13B, LEU14B, and CYS15B through alkyl and π–alkyl interactions. (C) Interaction map generated in Discovery Studio, confirming the same network of polar and hydrophobic interactions that stabilize the complex.

In addition, CR2106 exhibited poor binding energy (−8.0 kcal/mol), with superficial interactions restricted to peripheral polar loops. These contacts are unlikely to compromise channel structure or function, consistent with its lower activity against MTB. Collectively, these modeling studies support the hypothesis that AMP efficacy may involve a multifaceted mode of action that includes interactions with membrane-associated proteins. In particular, the ability to engage CpnT may induce intracellular stress by depriving the pathogen of essential nutrients such as iron^43,45^ while indirectly weakening its ability to maintain the integrity of the outer envelope.

Taken together, these findings suggest that high-affinity binding to nutrient channels such as CpnT complements membrane-disruptive activity by restricting iron uptake and weakening the bacterium’s adaptive response. Importantly, this dual interference may also sensitize the cell wall to further destabilization. Since the structural integrity of MTB depends heavily on its thick envelope—dominated by mycolic acids and maintained through the activity of biosynthetic enzymes such as Ag85B—we next investigated whether these AMPs might also target these intracellular pathways.^47^

It is important to note that these proteins are embedded within, or functionally associated with, the mycolic acid-rich outer membrane of MTB. While they are not structural lipids themselves, their activity occurs in the context of this hydrophobic barrier, which dominates envelope architecture. Thus, peptide engagement with MspA and CpnT reflects interactions taking place within the lipidic mycolic environment, whereas Ag85B directly catalyzes the transfer of mycolic acids to cell wall components. This dual perspective links protein obstruction with disruption of the biosynthetic machinery sustaining the mycolic acid envelope. Accordingly, we examined potential interactions with Ag85B to assess whether AMP binding could interfere with mycolic acid metabolism, thereby further compromising the mycobacterial envelope from within.

### 3.4 Targeting Mycolic Acid Biosynthesis: AMP Binding to Ag85B Undermines Cell Wall Integrity

The lipid-rich envelope of MTB not only contributes to intrinsic drug resistance but also anchors immunomodulatory molecules such as trehalose dimycolate.^48^ This barrier is assembled and maintained by the antigen 85 (Ag85) complex, a trio of essential mycolyltransferases.^48^ Among them, Ag85B catalyzes the transfer of mycolic acids to trehalose and arabinogalactan, playing a central role in cell wall biogenesis.^49^ We therefore explored, via molecular docking, whether AMP binding might interfere with this enzymatic machinery, potentially disrupting envelope formation (Figure 6). This molecular docking study revealed that W-B1CTcu5 exhibited a favorable interaction with Ag85B (−9.0 kcal/mol), positioning itself within the catalytic groove.^49^ In the comparative analysis of the two interaction models, eight residues were consistently identified as critical for complex stability. Specifically, Trp207 participates in hydrophobic π–alkyl contacts, Asn254 and Asn258 form stable hydrogen bonds, and Tyr209 and Tyr265 also establish hydrogen bonds reinforcing ligand orientation. Ala268 and Ala272 contribute additional hydrophobic contacts, while Gln269 maintains a direct hydrogen bond with the peptide. Together, these polar and hydrophobic interactions constitute the primary recognition core for W-B1CTcu5 binding to Ag85B (Figure 6).^49^

**Figure 6:**
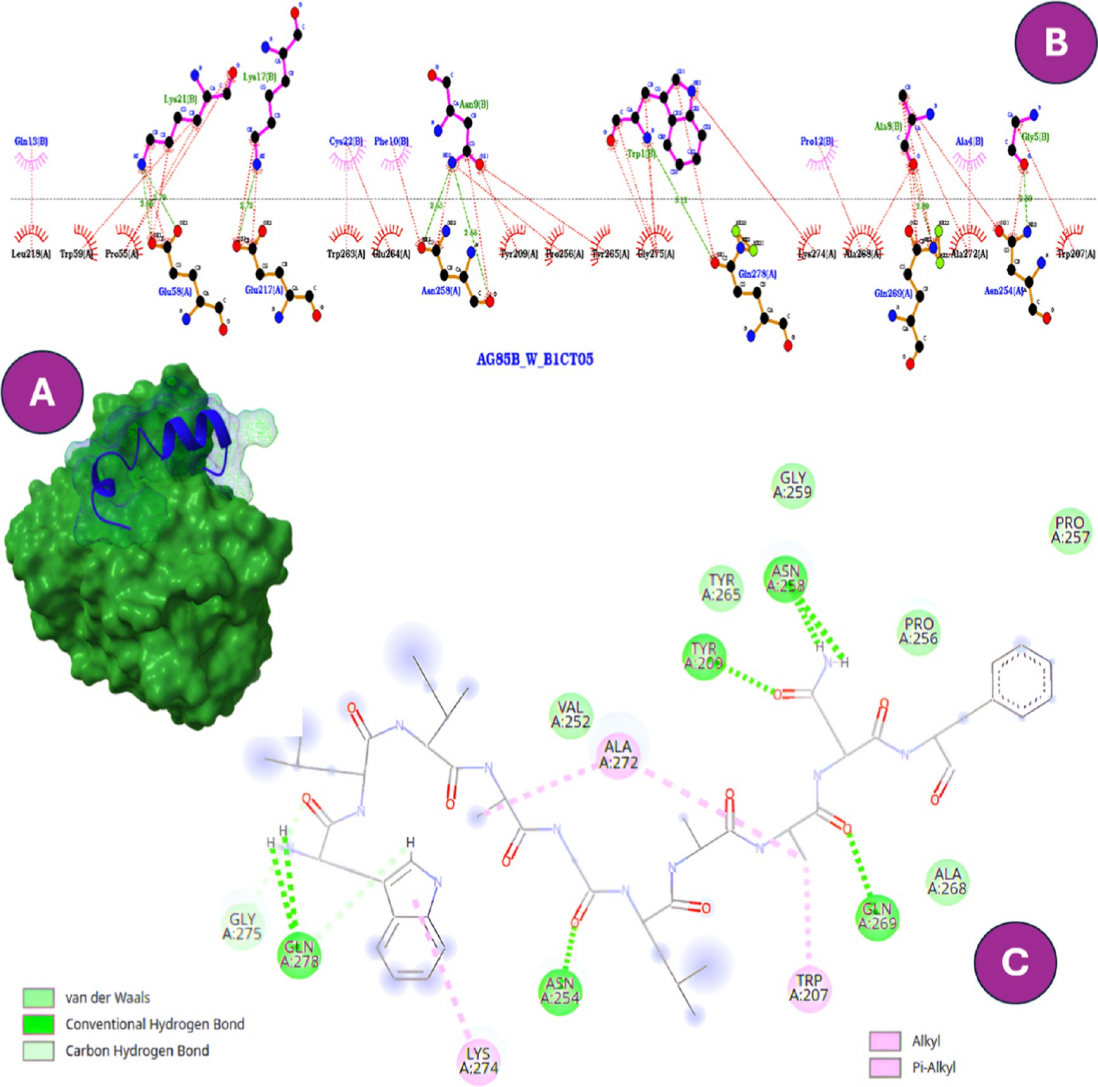
(A) Binding mode of W-B1CTcu5 with Ag85B. (B) Two-dimensional interaction map highlighting key residues (Trp207, Asn254, Asn258, Tyr209, Tyr265, Ala268, Ala272, and Gln269) involved in hydrogen bonding and hydrophobic contacts. (C) Same complex shown in Discovery Studio, confirming the stabilizing network of polar and hydrophobic interactions.

This molecular docking study revealed that W-B1CTcu5 exhibited a favorable interaction with Ag85B (−9.0 kcal/mol), positioning itself within the catalytic groove.^49^ In the comparative analysis of the two interaction models, eight residues were consistently identified as critical for complex stability. Specifically, Trp207 participates in hydrophobic π–alkyl contacts, Asn254 and Asn258 form stable hydrogen bonds, and Tyr209 and Tyr265 also establish hydrogen bonds reinforcing ligand orientation. Ala268 and Ala272 contribute additional hydrophobic contacts, while Gln269 maintains a direct hydrogen bond with the peptide. Together, these polar and hydrophobic interactions constitute the primary recognition core for W-B1CTcu5 binding to Ag85B (Figure 6).

CR2106 also engaged the catalytic region (−9.0 kcal/mol).^48^ The interaction network involved His261, Ser262, Trp263, Gln44, Arg42, Asn222, Ala225, Leu41, Ala166, and Leu228. Among these, Gln44, Arg42, Asn222, and His261 formed conventional hydrogen bonds, while Trp263 and Leu228 contributed aromatic and hydrophobic contacts favoring stacking and lateral packing. Leu41 and Ala166 provided further hydrophobic stabilization, collectively forming a multifunctional anchoring core that may interfere with Ag85B’s catalytic activity (Figure 7).

**Figure 7:**
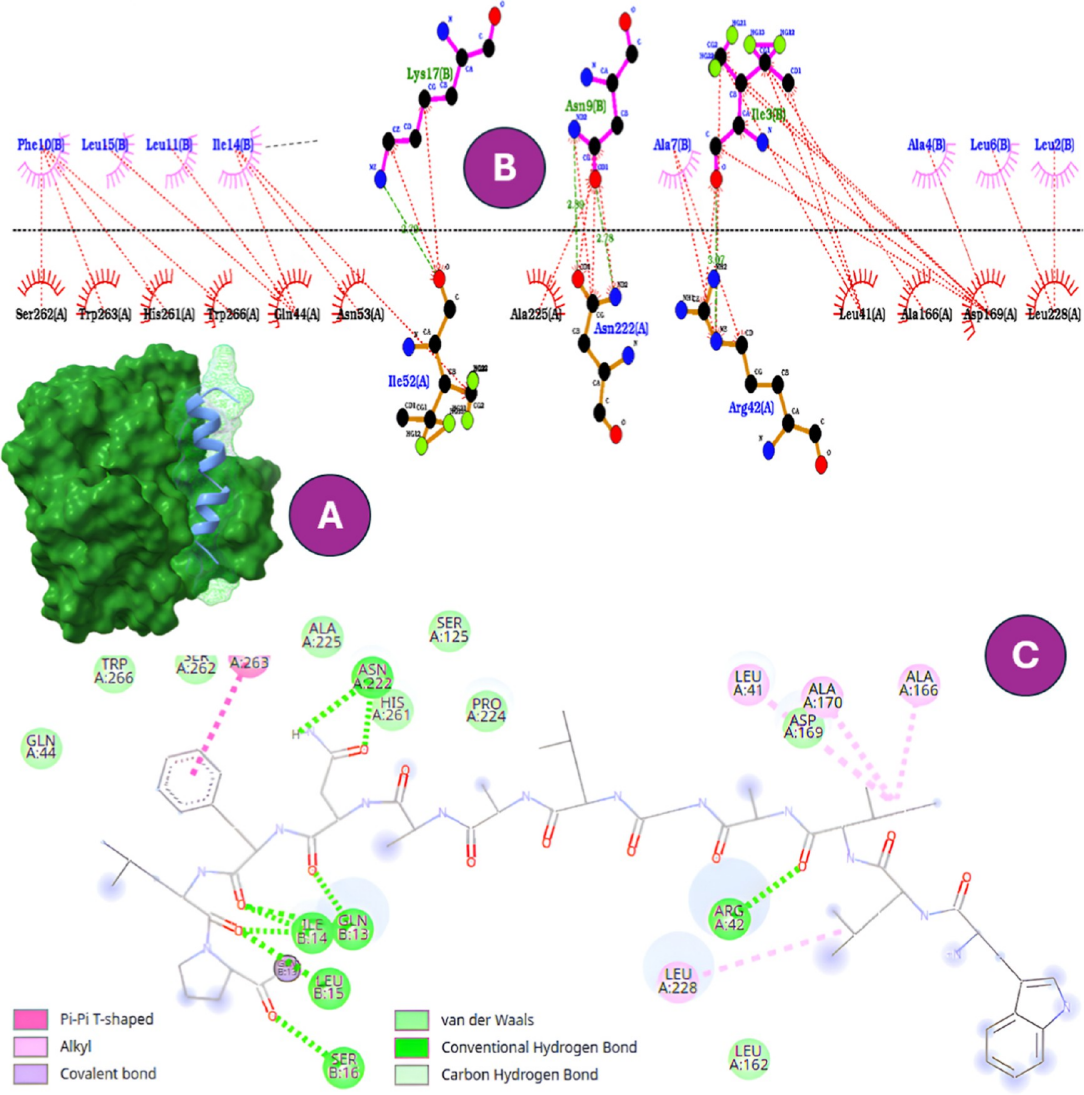
(A) Binding mode of CR2106 with Ag85B. (B) Two-dimensional interaction map highlighting key residues (His261, Ser262, Trp263, Gln44, Arg42, Asn222, Ala225, Leu41, Ala166, and Leu228). (C) Same complex shown in Discovery Studio, confirming the multifunctional anchoring network.

In contrast, B1CTcu5 and CR2111 docked to distal, nonfunctional regions with reduced affinities (−7.6 and −8.6 kcal/mol), supporting their lack of inhibition for this molecular target. These findings extend previously established structure–activity relationships: Trp insertion not only enhances membrane affinity and porin binding, but also increases the probability of interactions with intracellular enzymatic targets. Overall, these observations support a multifaceted mechanism, in which membrane disruption, porin interaction, and enzymatic engagement collectively compromise MTB viability. Importantly, these interactions appear to be modulated by physicochemical properties such as hydrophobicity, charge distribution, and secondary structure propensity.^47^ However, to fully capture peptide behavior in a biologically relevant context, particularly within mycolic acid–enriched membranes, a dynamic framework is required. Accordingly, molecular dynamics simulations were employed to refine mechanistic hypotheses beyond static docking models.

### 3.5 Membrane Engagement Dynamics Reveal Functional Divergence across Peptides

To move beyond static structural predictions and explore peptide–membrane interactions over biologically relevant time scales, we employed molecular dynamics (MD) simulations using a bilayer enriched in mycolic acid analogs designed to mimic the lipid complexity of the MTB outer membrane. This computational framework enabled assessment of orientation, insertion depth, conformational variability, and surface contact persistence—descriptors increasingly recognized as informative for predicting membrane activity.^50^ Nonetheless, our model represents a simplification of the MTB outer membrane which, while providing valuable mechanistic insights, does not capture its full chemical and structural diversity. In reality, the MTB envelope comprises not only α-mycolic acids but also ketomycolic acids, methoxymycolic acids, trehalose dimycolate, trehalose monomycolate, arabinogalactan–peptidoglycan, phthiocerol dimycocerosates, diacyl trehaloses, pentaacyl trehaloses, and sulfated trehalose glycolipids. Thus, restricting the model to the most abundant structural component constitutes an inherent limitation.^51,52^

Furthermore, relatively few MD studies have examined the interaction of the MTB outer membrane with antitubercular molecules. Modeling the cell wall still faces significant challenges, due to both the lack of standardized tools and the high computational costs required to incorporate its full molecular heterogeneity. As a result, most current studies focus on structural dynamics, thermodynamic properties, and conformational stability of membranes containing only mycolic acids.^53^ Within this context, our work represents the first attempt to elucidate AMP–bilayer interactions in MTB from a mechanistic perspective. Notably, studies such as that of Basu et al.,^10^ on which we based our choice of the GROMOS 54A7-ATB force field and a 100 ns production cycle,^54^ have primarily examined small molecules interacting with monolayers. The use of GROMOS 54A7-ATB is justified because it more consistently captures drug–mycolic acid interactions and better represents compound solubility in this environment. Consequently, our study provides a baseline framework for future models incorporating higher-order complexity, such as coarse-grained MARTINI simulations. These approaches will enable the capture of processes over longer time scales and provide insights from a multiscale perspective, contributing to a more complete understanding of AMP–membrane interactions. However, while coarse-grained models offer advantages in temporal scaling, they currently face limitations in accurately incorporating mycolic acid stereochemistry. The adoption of improved force fields for such analyses will therefore be addressed in future studies.

Among the tested analogs, W-B1CTcu5, the most potent in vitro, demonstrated prolonged electrostatic engagement at the bilayer interface and preferential alignment parallel to the membrane surface. This orientation is consistent with a nonlytic mechanism involving surface destabilization rather than full bilayer penetration. Its amphipathic character, reinforced by the N-terminal Trp residue, likely contributes to energetically favorable anchoring without inducing membrane rupture. CR2111 mirrored some of these traits, displaying moderate surface retention and localized interaction zones. Such restrained dynamics may enable transient associations with outer-membrane proteins, thereby supporting its balanced antimicrobial efficacy and selectivity profile. In contrast, CR2106 exhibited deeper insertion but unstable interactions, including bimodal center-of-mass fluctuations and conformational disorder. These features suggest less favorable or poorly sustained engagement, consistent with its comparatively lower activity. Finally, B1CTcu5 displayed only weak and transient contact with the bilayer, in agreement with its minimal antimicrobial effect and reduced membrane affinity.

Overall, the simulation-derived descriptors—namely insertion depth, contact stability, and dynamic persistence—provide a useful basis for distinguishing functional from nonfunctional peptide behavior. Although not definitive, these correlations highlight the value of membrane-focused MD in guiding AMP optimization.^50^ To complement these analyses, MD trajectories were visualized (Videos S1, S2, S3 and S4, Figures S2–S5), illustrating dynamic behavior including surface anchoring, transient embedding, and orientation shifts over the 100 ns time scale. These visualizations enhance mechanistic interpretation and may inform future refinements in AMP structural design.

### 3.6 Structural Dynamics: Interpreting RMSD and RMSF Profiles

RMSD and RMSF analyses were performed over 100 ns of MD simulations to evaluate the structural behavior of the peptides in a membrane-like environment (Figure 8).^55^ These descriptors capture both global conformational drift and local flexibility, and together they offer insight into structural persistence under physiological-like fluctuations—an essential parameter for membrane-active agents.^56–58^ Among the four peptides, CR2106 exhibited the highest RMSD (∼0.7 nm), with no indication of convergence across the trajectory (Figure 8A), suggesting an intrinsically disordered and conformationally unstable backbone. Its RMSF profile corroborates this dynamic instability, revealing widespread flexibility not only at the termini but also across central residues, particularly the LAANF motif (Figure 8B). This lack of structural constraint may explain its limited antimicrobial efficacy (MIC = 22.3 μg/mL), as excessive plasticity likely hinders the preservation of spatial pharmacophores essential for protein binding. Despite its ability to insert into the membrane core (see Section 3.7), its instability suggests a propensity for nonspecific interactions and loss of target recognition.

**Figure 8:**
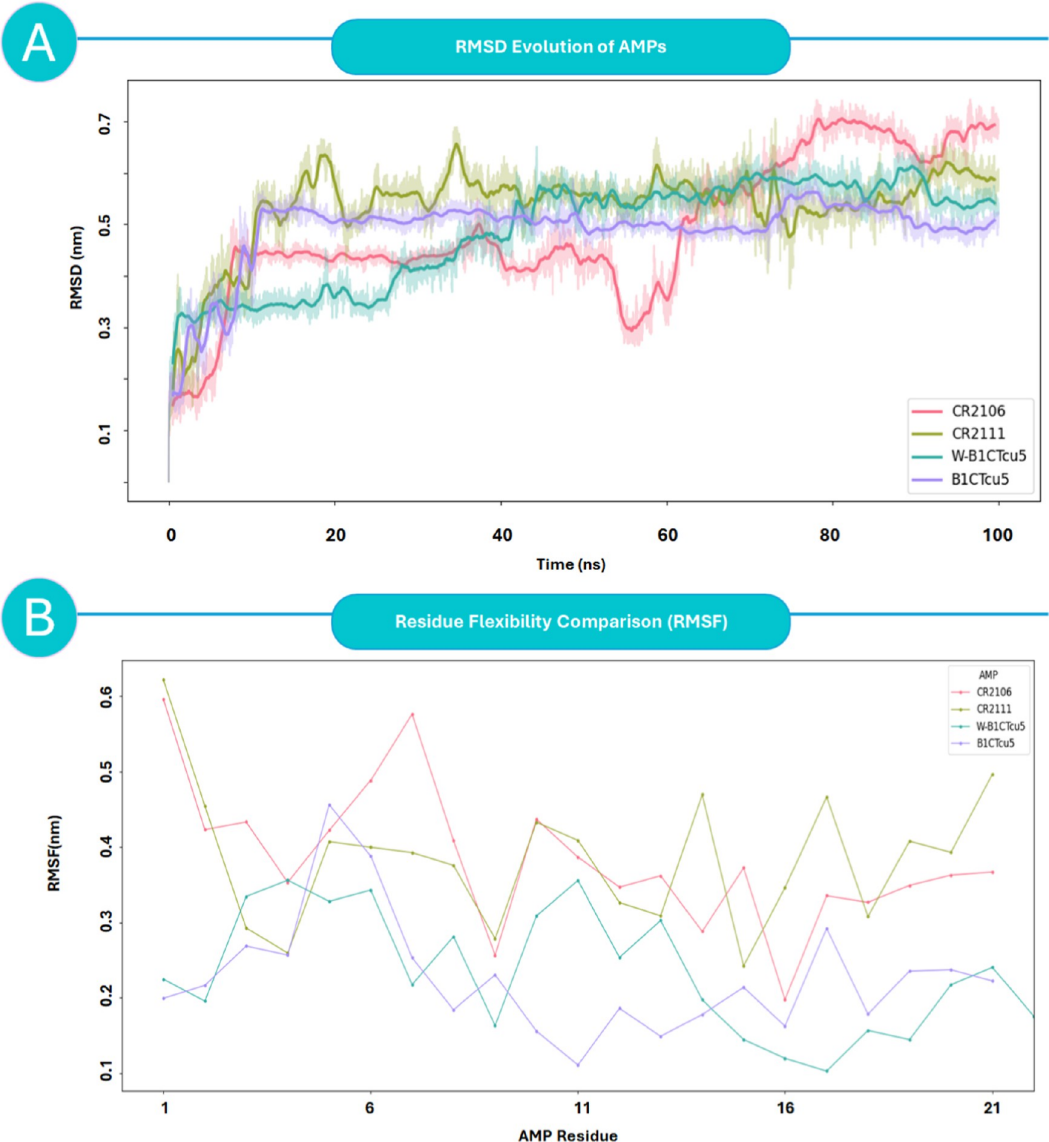
Conformational dynamics of AMPs during 100 ns molecular dynamics simulations. (A) Root-mean-square deviation (RMSD) profiles showing the global structural stability of each peptide. W-B1CTcu5 and B1CTcu5 maintain low and stable RMSD values (∼0.5 nm), indicating high conformational rigidity. CR2106 exhibits the highest RMSD (∼0.7 nm) with increasing deviation over time, consistent with structural disorder. CR2111 shows intermediate stability with mild fluctuations. (B) Per-residue root-mean-square fluctuation (RMSF) analysis highlighting local flexibility along the peptide sequence. CR2106 displays elevated flexibility across both termini and central residues, while W-B1CTcu5 exhibits the lowest fluctuations, particularly in the LAANFLPQILC core and Trp1 region. These trends support a link between conformational persistence and antimicrobial performance.

In contrast, W-B1CTcu5 displayed the most stable trajectory, with low RMSD (∼0.5 nm) and rapid convergence. Its per-residue fluctuation was minimal (<0.15 nm), particularly in the central LAANFLPQILC domain and at the N-terminal Trp1. This structural rigidity can be attributed to hydrophobic packing facilitated by Trp1, which appears to stabilize the helical core. Such persistence of a compact, amphipathic conformation under dynamic conditions may support sustained interactions with membrane-embedded proteins such as MspA, Ag85B, or CpnT. CR2111 presented an intermediate profile, with moderate RMSD values (∼0.6 nm) and a mixed rigidity–flexibility pattern. Its core residues (6–15) remained structurally stable, while both termini displayed moderate fluctuations. This balance may enable adaptive binding to membrane interfaces while maintaining a bioactive fold—consistent with its moderate MIC (7.4 μg/mL) and defined docking signatures.

Interestingly, B1CTcu5—although sharing sequence similarity with W-B1CTcu5—exhibited slightly elevated RMSF at the *N*-terminal region due to the absence of tryptophan. This subtle shift in packing stability may account for its reduced potency (MIC = 12.3 μg/mL), highlighting how even minimal sequence alterations can influence long-range conformational behavior. Taken together, these data reinforce a central principle: structural persistence under thermal and conformational noise correlates more strongly with bioactivity than membrane insertion alone. Peptides that maintain low conformational drift and localized flexibility are better suited for specific interactions with membrane receptors and are less likely to undergo degradation or off-target binding. However, this same rigidity may enhance membrane anchoring in host cells, suggesting a complex trade-off between stability, selectivity, and toxicity—a point further explored below.

### 3.7 Membrane Engagement: Interpreting COM Dynamics and Functional Outcomes

To complement the structural insights obtained from RMSD/RMSF, we analyzed the center-of-mass (COM) distances between each peptide and the bilayer surface throughout the simulation (Figure 9). This metric serves as a proxy for membrane insertion depth and spatiotemporal association patterns with lipid surfaces, particularly relevant in the context of mycolic acid-enriched bilayers. CR2106 displayed a progressive and unstable insertion pattern, with its COM shifting from ∼2.0 to ∼1.0 nm over time (Figure 9A). This downward trajectory reflects deep, dynamic penetration into the membrane core, likely driven by hydrophobic collapse rather than stable amphipathic alignment. The corresponding heatmap (Figure 9B) and bimodal distribution (Figure 9C) further suggest an inconsistent engagement profile, possibly reflecting conformational unfolding and helix-to-coil transitions during membrane translocation. While such behavior may transiently disrupt lipid order, it lacks the precision required for target-oriented antimicrobial activity—consistent with CR2106’s low efficacy and structural disorder.

**Figure 9:**
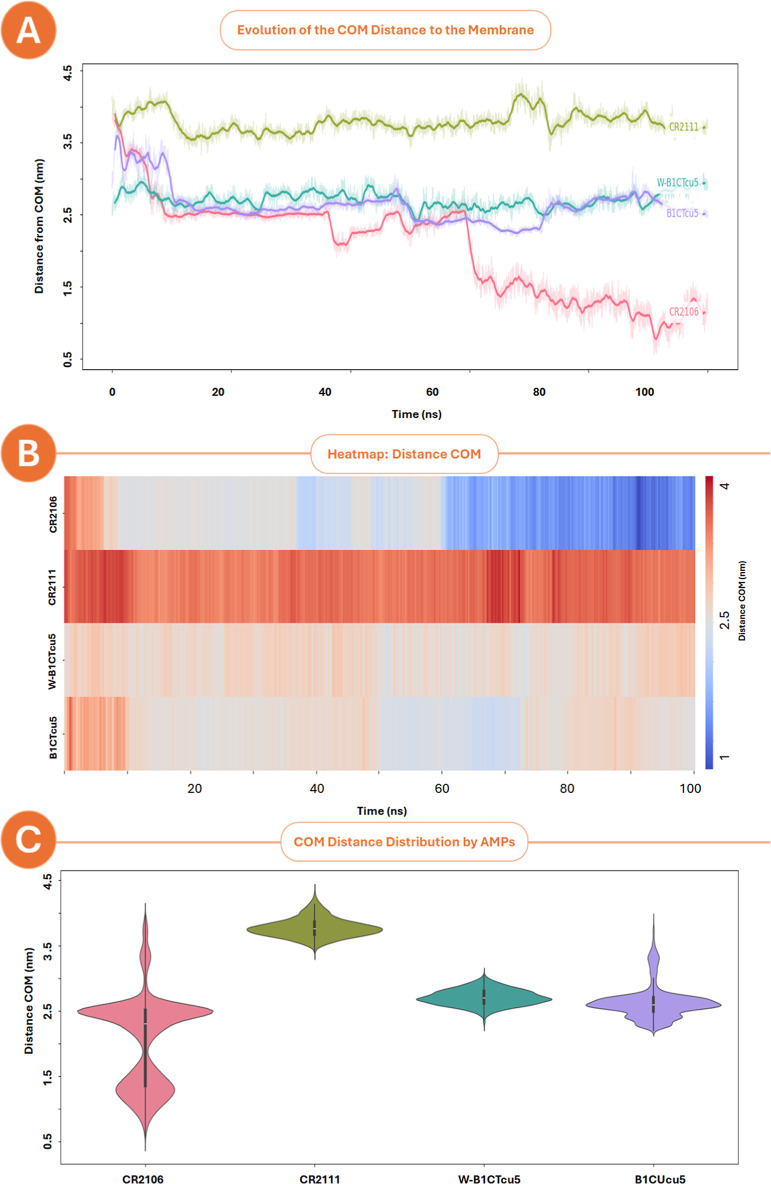
Center-of-Mass (COM) analysis of peptide–membrane interactions over 100 ns of MD simulations in a mycolic acid–enriched bilayer. (A) Time-resolved trajectories showing COM–bilayer distance. W-B1CTcu5 and B1CTcu5 exhibit stable surface–level association (∼2.5 nm), CR2106 shows progressive insertion toward the membrane core (∼1.0–2.0 nm), and CR2111 maintains the highest average distance (∼3.5–4.0 nm). (B) Heatmap visualization of COM distance per frame, highlighting temporal association patterns. CR2106 fluctuates with deeper penetration, while W-B1CTcu5 and B1CTcu5 remain consistently shallow. (C) Violin plots of COM distance distributions, illustrating density and variability of membrane engagement. The bimodal profile of CR2106 reflects dynamic instability, in contrast to the tighter distributions of W-B1CTcu5 and B1CTcu5.

By contrast, W-B1CTcu5 and B1CTcu5 remained anchored at ∼2.5 nm from the bilayer center, maintaining a shallow yet consistent membrane association. This positioning suggests surface alignment, compatible with stable amphipathic helix orientation and interaction with peripheral membrane proteins. CR2111 exhibited the highest average COM (>3.5 nm), indicating superficial contact and possible electrostatic interactions without significant bilayer penetration. These COM dynamics align closely with the structural analyses in Section 3.6 Peptides with high RMSD (e.g., CR2106) tended to insert deeply and erratically into the membrane, while those with low conformational drift (e.g., W-B1CTcu5) preserved peripheral anchoring. However, insertion depth alone did not correlate with potency—sustained conformational integrity during membrane engagement appears more critical for productive antimicrobial action.

This distinction has practical implications. Deep insertion may enhance membrane disruption but at the expense of specificity and structural fidelity. Shallow, stable anchoring—when paired with conformational rigidity—may favor selective docking to membrane proteins while minimizing off-target cytotoxicity. Yet even here, caution is warranted: W-B1CTcu5, despite its desirable biophysical profile, exhibited significant hemolytic activity (93.1% at 400 μg/mL), likely due to sustained amphipathic engagement with host membranes. This duality underscores the limitations of relying solely on membrane metrics to predict therapeutic index.

Importantly, the pronounced cytotoxicity and hemolysis observed for W-B1CTcu5 highlight a well-documented trade-off in AMP development, where enhanced amphipathicity boosts antimicrobial potency but compromises host cell selectivity. Similar outcomes have been reported for other Trp-rich analogues, underscoring that potency gains cannot be considered in isolation from toxicity liabilities. Several strategies could be envisioned to mitigate these effects, including incorporation of d-amino acids to reduce proteolysis and off-target binding, head-to-tail cyclization to restrict conformational flexibility, PEGylation or other masking approaches to attenuate nonspecific membrane disruption, and encapsulation into macrophage-targeted nanocarriers to reduce systemic exposure.^59,60^ These modifications, though beyond the scope of the present study, represent realistic avenues for improving the translational potential of W-B1CTcu5 and related analogues.

### 3.8 Limitations

First, the analogue W-B1CTcu5, which incorporates an additional tryptophan residue to enhance amphipathicity, exhibited significant fibroblast cytotoxicity and pronounced hemolysis (∼93% at 400 μg/mL). This trade-off between potency and host selectivity is a well-known barrier in AMP development and restricts the immediate translational applicability of this analogue. This study presents important insights but also has several limitations. Further structural modifications (e.g., incorporation of d-amino acids, cyclization, PEGylation) or encapsulation strategies (e.g., macrophage-targeted or pH-responsive nanocarriers) will be needed to mitigate these effects.

Second, our MD model employed only α-mycolic acids and did not incorporate keto- and methoxy-mycolates or other cell wall components, which may limit predictive accuracy. Third, although several protein targets were initially screened, only MspA, CpnT, and Ag85B with the highest predicted affinities were analyzed in detail; additional targets of therapeutic relevance remain unexplored. Finally, this study was restricted to in vitro assays; in vivo validation will be necessary to establish pharmacokinetics, immunological effects, and therapeutic safety. Altogether, these limitations highlight that while W-B1CTcu5 provides a valuable proof of concept, its current toxicity profile restricts systemic use. At the same time, these challenges open realistic avenues for optimization, where rational chemical modifications and advanced delivery platforms may bridge the gap between in vitro potency and clinical feasibility.

## 4 Conclusions and Outlooks

This study shows how combining molecular modeling with biological assays can provide a clearer view of how small sequence changes alter the behavior of antimicrobial peptides against MTB. By linking structural dynamics, docking profiles, and in vitro activity, we were able to test whether computational predictions truly reflect biological outcomes. The results suggest that stability parameters such as RMSD/RMSF and binding affinity may help anticipate antimicrobial performance, but also reveal that they cannot alone predict safety. Among the analogues examined, W-B1CTcu5 stood out for its potency, low MIC values, and stable interactions with key mycobacterial proteins. At the same time, its high hemolytic activity highlights the persistent problem of balancing efficacy with host compatibility. This finding underlines the importance of early recognition of toxicity, as well as the need to adapt the peptide scaffold through chemical modifications or targeted delivery systems. The membrane model was restricted to α-mycolic acids, and docking analyses focused only on the highest-affinity receptors. Moreover, all validation was performed in vitro, and the behavior of these peptides under host-like conditions or in infection models remains unresolved. Taken together, these results illustrate both the promise and the constraints of peptide design against tuberculosis. The integrative approach applied here may serve as a starting point for refining candidate molecules, guiding the next steps toward formulations that retain activity while addressing toxicity and stability in vivo.

## Supplementary Material











## Notes

MD simulation trajectory of B1CTcu5 in mycolic acid bilayer (100 ns) (MP4)
MD simulation trajectory of W-B1CTcu5 in mycolic acid bilayer (100 ns) (MP4)
MD simulation trajectory of CR2106 in mycolic acid bilayer (100 ns) (MP4)
MD simulation trajectory of CR2111 in mycolic acid bilayer (100 ns) (MP4)
Table S1, binding affinity and interaction contacts of AMP–protein complexes; Table S2, interaction profiles of receptor–AMP complexes predicted by PLIP; Table S3, interaction profiles of receptor–AMP complexes predicted by Discovery Studio; Figure S2, Ramachandran plots of peptide backbones after 100 ns MD simulation; Figure S3, final conformation of B1CTcu5 after 100 ns MD simulation in mycolic acid bilayer; Figure S4, final conformation of W-B1CTcu5 after 100 ns MD simulation in mycolic acid bilayer; Figure S5, final conformation of CR2106 after 100 ns MD simulation in mycolic acid bilayer; Figure S6, final conformation of CR2111 after 100 ns MD simulation in mycolic acid bilayer (PDF)
