# Supplementary material for: Integrating Docking, Dynamics, and Assays to Predict Antimicrobial Peptide Interactions with Mycolic Acid Membranes in Mycobacterium tuberculosis
Source: ACS Meas Sci Au. 2025 Oct 14;5(6):981–1000. doi: 10.1021/acsmeasuresciau.5c00126 (PMC12715740; doi:10.1021/acsmeasuresciau.5c00126)
Supplement: Supplementary file 5 [file tg5c00126_si_005.pdf]

Integrating docking, dynamics, and assays to predict antimicrobial peptide interactions with mycolic acid membranes in *Mycobacterium tuberculosis*

Cesar Augusto Roque-Borda[1,2,*,¥]; Oswaldo Julio Ramirez Delgado[1,¥]; Laura Maria Duran Gleriani Primo[1]; Emma Dyhr[3], Ingvill Pedersen Sæbø[4], Emily Helgesen[4,5], James Booth[4,5], Henrik Franzyk[3], Paul R Hansen[3], Hernan Morales-Navarrete[6]; Beatriz G. de la Torre[7,8]; Fernando Albericio[8,9]; João Perdigão[2]; Fernando Rogério Pavan[1*].

[1] Department of Biological Sciences, School of Pharmaceutical Sciences, Universidade Estadual Paulista (UNESP), 14800901, Araraquara, Brazil

[2] iMed.ULisboa–Institute for Medicines Research, Faculty of Pharmacy, University of Lisbon, 1649004 Lisbon, Portugal.

[3] University of Copenhagen, Faculty of Health and Medical Sciences, Department of Drug Design and Pharmacology, Copenhagen, 2100, Denmark.

[4] Department of Microbiology, Oslo University Hospital and the University of Oslo, Rikshospitalet, 0373 Oslo, Norway.

[5] Department of Clinical and Molecular Medicine, Norwegian University of Science and Technology, and Clinic of Laboratory Medicine, St. Olavs Hospital, 7491 Trondheim, Norway.

[6] Bio-Cheminformatics Research Group, Universidad de Las Américas, Quito, 170504, Ecuador.

[7] School of Laboratory Medicine and Medical Sciences, College of Health Sciences, University of KwaZulu-Natal, Durban, 4041, South Africa.

[8] Peptide Science Laboratory, School of Chemistry and Physics, University of KwaZulu-Natal, Durban 4001, South Africa

[9] Department of Organic Chemistry, University of Barcelona, 08028 Barcelona, Spain

[¥] These authors contributed equally to this work

Correspondence

* Cesar Augusto Roque-Borda, School of Pharmaceutical Sciences, Araraquara, São Paulo, – Brazil and iMed.ULisboa–Institute for Medicines Research, Faculty of Pharmacy, University of Lisbon, 1649004 Lisbon, Portugal. E-mail: cesar.roque@unesp.br.

* Fernando Rogério Pavan, São Paulo State University (UNESP), School of Pharmaceutical Sciences, Araraquara, São Paulo, – Brazil. E-mail: fernando.pavan@unesp.br

Table S1. Detailed information on binding affinity, dissociation constant, number and type of intermolecular contacts within a 5.5 Å cutoff.

Receptor	Ligand	Affinity (kcal/mol)	K_d (M)	ICs c-c	ICs c-p	ICs c-a	ICs p-p	ICs p-a	ICs a-a	NIS c	NIS a
	B1CTcu5	-6.1	5.4e- 05	2	0	11	0	7	13	27.31	48.15
**PE/PPE**	W- B1CTcu5	-7.3	7.1e-06	3	2	12	1	12	9	27.1	47.66
	CR2106	-7.8	3e-06	4	1	10	0	14	15	27.78	47.22
	CR2111	-7.6	4.6e-06	2	3	14	0	12	26	27.7	47.42
	B1CTcu5	-8.3	1.4e-06	3	6	12	0	14	24	17.61	52.83
**Porin MSPA**	W- B1CTcu5	-9.6	1.8e-07	1	2	14	3	22	28	17.72	52.53
	CR2106	-10.1	8.1e-08	3	5	17	1	20	26	17.14	52.7
	CR2111	-9.1	3.7e-07	0	2	15	1	19	27	17.72	53.48
	B1CTcu5	-9.4	2.2e-07	1	3	26	2	17	44	19.79	53.21
**CpnT**	W-B1CTcu5	-11.3	1e-08	8	5	21	1	24	30	19.65	53.61
	CR2106	-8.0	2.4e-06	7	7	13	1	13	36	19.52	53.52
	CR2111	-5.6	0.00012	0	1	6	3	10	37	19.6	53.33
	B1CTcu5	-6.8	1.5e-05	1	1	8	0	9	19	22.53	47.83
**Rv1698**	W- B1CTcu5	-7.8	3e-06	0	0	22	1	9	21	22.05	48.82
	CR2106	-7.8	3.3e-06	3	5	14	0	9	25	22.53	47.04
	CR2111	-8.3	1.5e-06	0	1	11	0	15	18	22.92	48.22
	B1CTc5	-7.6	4.7e-06	0	1	9	0	7	25	17.45	45.28
**Ag85B**	W-B1CTcu5	-9.0	4.7e-07	3	5	6	4	18	23	18.05	46.34
	CR2106	-9.0	4.7e-07	2	3	13	4	15	17	17.56	46.34
	CR2111	-8.6	9.1e-07	1	1	3	0	15	29	17.79	46.63

ΔG (kcal mol⁻¹): Gibbs free energy change of the protein-protein complex (negative values indicate favorable binding). **Kd (M) at ℃**: Dissociation constant at a specific temperature (lower Kd = higher affinity). **ICs charged-charged**: Interaction contacts between charged residues (e.g., Lys-Asp). **ICs charged-polar**: Contacts between charged and polar residues (e.g., Arg-Ser). **ICs charged-apolar**: Contacts between charged and hydrophobic residues (e.g., Glu-Ala). **ICs polar-polar**:

Contacts between polar residues (e.g., Thr-Asn). **ICs polar-apolar**: Contacts between polar and hydrophobic residues (e.g., Gln-Val). **ICs apolar-apolar**: Hydrophobic interactions (e.g., Leu-Ile). **NIS charged**: Non-interacting charged residues (solvent-exposed). **NIS apolar**: Non-interacting hydrophobic residues.

Table S2. Detailed information on the interaction profile of Receptor-AMP complexes predicted by Protein-Ligand Interaction Profiler (PLIP).

Receptor	Ligand	Interaction Type	Protein Residue	Ligand Residue	Distance (Å)	Notes
**PorinMspA (A)**	W- B1CTcu5	Hydrophobic Interaction	HIS148 (A)	ILE3 (C)	3.72	Aromatic-Aliphatic
**PorinMspA (A)**	W- B1CTcu5	Hydrophobic Interaction	VAL128 (A)	LEU6 (C)	3.72	Aliphatic-Aliphatic
**PorinMspA (A)**	W- B1CTcu5	Hydrophobic Interaction	VAL151 (A)	PHE10 (C)	3.63	Aliphatic-Aromatic
**PorinMspA (A)**	W- B1CTcu5	Hydrophobic Interaction	PRO123 (A)	PHE10 (C)	3.68	Aliphatic-Aromatic
**PorinMspA (A)**	W- B1CTcu5	Hydrophobic Interaction	THR152 (A)	LEU11 (C)	3.85	Polar-Aliphatic
**PorinMspA (A)**	W- B1CTcu5	Hydrophobic Interaction	THR152 (A)	ILE14 (C)	3.62	Polar-Aliphatic
**PorinMspA (A)**	W- B1CTcu5	Hydrophobic Interaction	ASP118 (A)	LYS17 (C)	3.91	Charged-Charged
**PorinMspA (A)**	W- B1CTcu5	Hydrogen Bond	GLN126 (A)	ASN9 (C)	1.86	Backbone Donor
**PorinMspA (A)**	W- B1CTcu5	Hydrogen Bond	THR152 (A)	GLN13 (C)	1.85	Backbone Donor
**PorinMspA (A)**	W- B1CTcu5	Hydrogen Bond	SER73 (A)	GLN13 (C)	1.86	Sidechain Acceptor
**PorinMspA (A)**	W- B1CTcu5	Hydrogen Bond	ASP118 (A)	LYS17 (C)	1.78	Backbone Donor
**PorinMspA (B)**	W- B1CTcu5	Hydrophobic Interaction	GLN126 (B)	TRP1 (C)	3.50	Polar-Aromatic
**PorinMspA (B)**	W- B1CTcu5	Hydrophobic Interaction	ALA129 (B)	LEU2 (C)	3.86	Aliphatic-Aliphatic
**PorinMspA (B)**	W- B1CTcu5	Hydrogen Bond	GLU127 (B)	TRP1 (C)	2.06	Backbone Donor
**PorinMspA (B)**	W- B1CTcu5	Hydrogen Bond	GLU127 (B)	LEU2 (C)	2.50	Backbone Donor
**PorinMspA (B)**	W- B1CTcu5	Hydrogen Bond	ASN121 (B)	ILE14 (C)	2.51	Backbone Donor
**PorinMspA (A)**	CR2106	Hydrophobic Interaction	VAL128 (A)	ILE3 (C)	3.47	Aliphatic-Aliphatic
**PorinMspA (A)**	CR2106	Hydrophobic Interaction	VAL128 (A)	ILE3 (C)	3.47	Aliphatic-Aliphatic
**PorinMspA (A)**	CR2106	Hydrophobic Interaction	THR152 (A)	LEU15 (C)	3.89	Polar-Aliphatic
**PorinMspA (B)**	CR2106	Hydrophobic Interaction	PHE163 (B)	TRP1 (C)	3.52	Aromatic-Aromatic
**PorinMspA (B)**	CR2106	Hydrophobic Interaction	ARG165 (B)	TRP1 (C)	2.73	Charged-Aromatic
**PorinMspA (B)**	CR2106	Hydrophobic Interaction	ARG165 (B)	TRP1 (C)	2.30	Charged-Aromatic
**PorinMspA (B)**	CR2106	Hydrogen Bond	GLU63 (B)	TRP1 (C)	1.92	Sidechain Donor
**PorinMspA (B)**	CR2106	Hydrogen Bond	GLU127 (B)	TRP1 (C)	1.89	Backbone Donor

**PorinMspA (B)**	CR2106	Hydrogen Bond	SER73 (B)	ARG19 (C)	3.55	Sidechain Donor
**PorinMspA (B)**	CR2106	Hydrogen Bond	GLY122 (B)	ARG19 (C)	1.97	Backbone Donor
**PorinMspA (B)**	CR2106	pi-Cation Interaction	ARG165 (B)	TRP1 (C)	3.83	Cation - Aromatic
**PorinMspA (A)**	CR2111	Hydrophobic Interaction	VAL144 (A)	ILE2 (C)	3.95	Aliphatic-Aliphatic
**PorinMspA (A)**	CR2111	Hydrophobic Interaction	ALA147 (A)	ILE2 (C)	3.83	Aliphatic-Aliphatic
**PorinMspA (A)**	CR2111	Hydrophobic Interaction	THR152 (A)	LEU14 (C)	3.87	Polar-Aliphatic
**PorinMspA (A)**	CR2111	Hydrophobic Interaction	THR152 (A)	ILE17 (C)	3.73	Polar-Aliphatic
**PorinMspA (B)**	CR2111	Hydrophobic Interaction	ILE167 (B)	LEU1 (C)	3.89	Aliphatic-Aliphatic
**PorinMspA (A)**	CR2111	Hydrogen Bond	SER73 (A)	LYS20 (C)	2.83	Sidechain Acceptor
**PorinMspA (A)**	CR2111	Hydrogen Bond	VAL76 (A)	ALA21 (C)	3.97	Backbone Donor
**PorinMspA (B)**	CR2111	Hydrogen Bond	ASN121 (B)	ALA21 (C)	2.81	Sidechain Donor
**PorinMspA (B)**	CR2111	Hydrogen Bond	SER173 (B)	LEU1 (C)	2.85	Sidechain Acceptor
**CpnT**	B1CTu5	Hydrophobic	VAL380 (A)	ILE2 (B)	3.96	Close hydrophobic contact
**CpnT**	B1CTu5	Hydrophobic	VAL51 (A)	ILE2 (B)	3.76	Hydrophobic cluster
**CpnT**	B1CTu5	Hydrophobic	VAL51 (A)	ALA3 (B)	3.82	Hydrophobic cluster
**CpnT**	B1CTu5	Hydrophobic	TRP384 (A)	LEU5 (B)	3.86	Pi interaction possible nearby
**CpnT**	B1CTu5	Hydrophobic	ALA50 (A)	ALA7 (B)	3.44	Strong hydrophobic contact
**CpnT**	B1CTu5	Hydrophobic	TRP397 (A)	ASN8 (B)	3.95	Hydrophobic sidechain
**CpnT**	B1CTu5	Hydrophobic	ILE392 (A)	PHE9 (B)	3.62	Multiple aromatic contacts
**CpnT**	B1CTu5	Hydrophobic	ILE392 (A)	PHE9 (B)	3.90	Multiple aromatic contacts
**CpnT**	B1CTu5	Hydrophobic	ILE392 (A)	PHE9 (B)	3.99	Multiple aromatic contacts
**CpnT**	B1CTu5	Hydrophobic	TRP384 (A)	LEU10 (B)	3.39	Close hydrophobic interaction
**CpnT**	B1CTu5	Hydrophobic	PHE387 (A)	LEU10 (B)	3.90	Pi stacking potential
**CpnT**	B1CTu5	Hydrogen Bond	ASP278 (A)	LEU1 (B)	2.67	Backbone H-bond
**CpnT**	B1CTu5	Hydrogen Bond	ASP278 (A)	ILE2 (B)	2.95	Sidechain H-bond
**CpnT**	B1CTu5	Salt Bridge	ASP57 (A)	LYS20 (B)	5.20	Electrostatic salt bridge
**CpnT**	B1CTu5	Pi-Stacking	TRP397 (A)	PHE9 (B)	5.06	T-shaped aromatic stacking

**CpnT**	B1CTu5	Pi-Stacking	TRP397 (A)	PHE9 (B)	5.21	T-shaped aromatic stacking
**CpnT (A)**	W-B1CTcu5 (B)	Hydrophobic	PRO 137	GLN 13	3.59	Favorable nonpolar contact.
**CpnT (A)**	W-B1CTcu5 (B)	Hydrophobic	TRP 669	LYS 21	3.79	Lateral hydrophobic interaction.
**CpnT (A)**	W-B1CTcu5 (B)	Hydrophobic	PRO 719	ALA 8	3.86	Stable aliphatic contact.
**CpnT (A)**	W-B1CTcu5 (B)	Hydrophobic	TYR 724	PRO 12	3.50	Aromatic-proline interaction.
**CpnT (A)**	W-B1CTcu5 (B)	Hydrophobic	PHE 727	LEU 15	3.82	Aromatic-aliphatic stacking.
**CpnT (A)**	W-B1CTcu5 (B)	Hydrophobic	PHE 727	LEU 15	3.99	Repeated contact, borderline range.
**CpnT (A)**	W-B1CTcu5 (B)	Hydrophobic	TYR 765	ILE 18	3.52	Aliphatic-aromatic contact.
**CpnT (A)**	W-B1CTcu5 (B)	Hydrogen Bond	GLY 133	ASN 9	3.00	Backbone H-bond.
**CpnT (A)**	W-B1CTcu5 (B)	Hydrogen Bond	PRO 137	LYS 17	2.82	Ligand as donor.
**CpnT (A)**	W-B1CTcu5 (B)	Hydrogen Bond	LYS 245	CYS 22	2.74	Strong sidechain H-bond.
**CpnT (A)**	W-B1CTcu5 (B)	Hydrogen Bond	GLN 660	TRP 1	2.78	Ligand donates H-bond.
**CpnT (A)**	W-B1CTcu5 (B)	Hydrogen Bond	GLN 722	ASN 9	2.86	Dual donor sidechains.
**CpnT (A)**	W-B1CTcu5 (B)	Hydrogen Bond	GLN 723	LYS 21	2.76	Protein acts as donor.
**CpnT (A)**	W-B1CTcu5 (B)	Hydrogen Bond	ARG 757	ILE 18	3.02	Reinforced H-bond.
**CpnT (A)**	W-B1CTcu5 (B)	Hydrogen Bond	ARG 757	ILE 18	2.84	Consecutive H-bond.
**CpnT (A)**	W-B1CTcu5 (B)	Salt Bridge	ASP 253	ARG 20	3.68	Strong ionic interaction.
**CpnT (A)**	W-B1CTcu5 (B)	Salt Bridge	ASP 789	ARG 20	3.33	Additional salt bridge.
**CpnT (A)**	W-B1CTcu5 (B)	pi-Cation Interaction	TYR 138	LYS 17	4.05	Aromatic ring stabilizes cation.
**CpnT (A)**	W-B1CTcu5 (B)	pi-Cation Interaction	TRP 669	LYS 21	2.96	Strong pi-cation interaction.
**Ag85B (A)**	W-B1CTcu5 (B)	Hydrophobic	PRO 55	LYS 21	3.98	Hydrophobic contact at proline ring.
**Ag85B (A)**	W-B1CTcu5 (B)	Hydrophobic	PRO 55	LYS 21	3.80	Second contact; same residues, different atoms.
**Ag85B (A)**	W-B1CTcu5 (B)	Hydrophobic	LEU 218	GLN 13	3.88	Favorable leucine–glutamine contact.
**Ag85B (A)**	W-B1CTcu5 (B)	Hydrophobic	ALA 268	PRO 12	3.83	Compact aliphatic interaction.
**Ag85B (A)**	W-B1CTcu5 (B)	Hydrophobic	ALA 272	ALA 8	3.41	Symmetric small-residue interaction.
**Ag85B (A)**	W-B1CTcu5 (B)	Hydrogen bond	TYR 209	ASN 9	3.49	H-bond via hydroxyl and carbonyl oxygen.

**Ag85B (A)**	W-B1CTcu5 (B)	Hydrogen bond	ASN 216	GLN 13	4.07	Long-range polar interaction.
**Ag85B (A)**	W-B1CTcu5 (B)	Hydrogen bond	ASN 254	GLY 5	2.89	Strong backbone-mediated H-bond.
**Ag85B (A)**	W-B1CTcu5 (B)	Hydrogen bond	ASN 258	ASN 9	2.88	Polar interaction between side chains.
**Ag85B (A)**	W-B1CTcu5 (B)	Hydrogen bond	GLN 269	ALA 8	2.89	Amide–carbonyl H-bond.
**Ag85B (A)**	W-B1CTcu5 (B)	Hydrogen bond	GLN 278	TRP 1	3.12	Moderate polar contact.
**Ag85B (A)**	W-B1CTcu5 (B)	Salt bridge	GLU 58	LYS 21	2.50	Strong electrostatic interaction.
**Ag85B (A)**	W-B1CTcu5 (B)	Salt bridge	GLU 217	LYS 17	3.26	Weaker but stabilizing interaction.
**Ag85B (A)**	CR2106 (B)	Hydrophobic	LEU 41	ILE 3	3.70	Aliphatic sidechain contact
**Ag85B (A)**	CR2106 (B)	Hydrophobic	ILE 52	LYS 17	3.93	Non-polar sidechain contact
**Ag85B (A)**	CR2106 (B)	Hydrophobic	ILE 52	ILE 14	3.55	Isoleucine-aliphatic contact
**Ag85B (A)**	CR2106 (B)	Hydrophobic	ALA 166	ILE 3	3.80	Small stable hydrophobic contact
**Ag85B (A)**	CR2106 (B)	Hydrophobic	LEU 228	LEU 6	3.57	Aliphatic chain interaction
**Ag85B (A)**	CR2106 (B)	Hydrophobic	LEU 228	LEU 2	3.85	Moderate hydrophobic contact
**Ag85B (A)**	CR2106 (B)	Hydrophobic	TRP 266	PHE 10	3.77	Aromatic sidechain contact
**Ag85B (A)**	CR2106 (B)	Hydrogen Bond	ARG 42	ILE 3	3.07	First bifurcated H-bond from guanidinium group
**Ag85B (A)**	CR2106 (B)	Hydrogen Bond	ARG 42	ILE 3	3.64	Second bifurcated H-bond from guanidinium group
**Ag85B (A)**	CR2106 (B)	Hydrogen Bond	ILE 52	LYS 17	2.79	Backbone H-bond
**Ag85B (A)**	CR2106 (B)	Hydrogen Bond	ASN 222	ASN 9	2.78	Mutual sidechain H-bond
**Ag85B (A)**	CR2106 (B)	Hydrogen Bond	ASN 222	ASN 9	2.89	Mutual sidechain H-bond
**Ag85B (A)**	CR2106 (B)	π-Stacking (T)	TRP 263	PHE 10	4.41	T-shaped aromatic π-π stacking

Table S3. Detailed information on the interaction profile of Receptor-AMP complexes predicted by Discover Studio.

Receptor	Ligand	Interaction Type	Protein Residue
**PorinMspA (A)**	W- B1CTcu5	Conventional Hydrogen Bond	THR152 (A)
**PorinMspA (A)**	W- B1CTcu5	Conventional Hydrogen Bond	SER73(A)
**PorinMspA (A)**	W- B1CTcu5	Conventional Hydrogen Bond	GLN126 (A)
**PorinMspA (A)**	W- B1CTcu5	Alkyl	VAL151 (A)
**PorinMspA (A)**	W- B1CTcu5	Pi-Alkyl	PHE70 (A)
**PorinMspA (A)**	W- B1CTcu5	Alkyl	PRO123 (A)
**PorinMspA (A)**	W- B1CTcu5	Alkyl	HIS148 (A)
**PorinMspA (A)**	W- B1CTcu5	Alkyl	ALA129 (B)
**PorinMspA (A)**	W- B1CTcu5	Conventional Hydrogen Bond	GLU127(B)
**PorinMspA (A)**	CR2106	Alkyl	VAL128 (A)
**PorinMspA (A)**	CR2106	Salt bridge	GLU127 (B)
**PorinMspA (A)**	CR2106	Unfavorable Donor-Donor	ARG165 (B)
**PorinMspA (B)**	CR2106	Pi-Pi T-shaped	PHE163 (B)
**PorinMspA (B)**	CR2106	Conventional Hydrogen Bond	GLU63 (B)
**PorinMspA (A)**	CR2111	Alkyl	ILE167 (B)
**PorinMspA (A)**	CR2111	Conventional Hydrogen Bond	SER173 (B)
**PorinMspA (A)**	CR2111	Conventional Hydrogen Bond	ASP172 (B)
**PorinMspA (A)**	CR2111	Conventional Hydrogen Bond	GLY171 (B)
**PorinMspA (B)**	CR2111	Carbon Hydrogen bond	THR130 (A)
**PorinMspA (A)**	CR2111	Alkyl	ALA147 (A)
**CpnT**	B1CTu5	Alkyl	VAL122 (A)
**CpnT**	B1CTu5	Alkyl	PHE387 (A)
**CpnT**	B1CTu5	Conventional Hydrogen Bond	ILE (B)
**CpnT**	B1CTu5	Alkyl	ALA50(A)

**CpnT**	B1CTu5	Conventional Hydrogen Bond	ASP45 (A)
**CpnT**	B1CTu5	Conventional Hydrogen Bond	ARG54 (A)
**CpnT**	B1CTu5	Alkyl	VAL51 (A)
**CpnT**	B1CTu5	Salt Bridge	ASP278 (A)
**CpnT**	B1CTu5	Conventional Hydrogen Bond	ASP278 (A)
**CpnT**	B1CTu5	Pi-Alkyl	TRP348 (A)
**CpnT**	B1CTu5	Pi-Pi T-shaped	TRP397 (A)
**CpnT**	B1CTu5	Alkyl	ILE392 (A)
**CpnT**	B1CTu5	Conventional Hydrogen Bond	CYS15 (B)
**CpnT**	B1CTu5	Conventional Hydrogen Bond	LEU14 (B)
**CpnT**	B1CTu5	Conventional Hydrogen Bond	LYS16 (B)
**CpnT**	W-B1CTcu5	Conventional Hydrogen Bond	ILE14 (B)
**CpnT**	W-B1CTcu5	Conventional Hydrogen Bond	GLN13 (B)
**CpnT**	W-B1CTcu5	Conventional Hydrogen Bond	GLY133 (A)
**CpnT**	W-B1CTcu5	Conventional Hydrogen Bond	GLN722 (A)
**CpnT**	W-B1CTcu5	Conventional Hydrogen Bond	GLN660 (A)
**CpnT**	W-B1CTcu5	Salt bridge	ASP658 (A)
**CpnT**	W-B1CTcu5	Alkyl	ALA136 (A)
**Ag58B**	W-B1CTcu5 (B)	Conventional Hydrogen Bond	ASN258 (A)
**Ag58B**	W-B1CTcu5 (B)	Conventional Hydrogen Bond	TYR209 (A)
**Ag58B**	W-B1CTcu5 (B)	Alkyl	ALA272 (A)
**Ag58B (A)**	W-B1CTcu5 (B)	Conventional Hydrogen Bond	GLN278 (A)
**Ag58B (A)**	W-B1CTcu5 (B)	Alkyl	LYS274 (A)
**Ag58B (A)**	W-B1CTcu5 (B)	Conventional Hydrogen Bond	ASN254 (A)
**Ag58B (A)**	W-B1CTcu5 (B)	Alkyl	TRP207 (A)
**Ag58B (A)**	CR2106 (B)	Alkyl	ALA166 (A)
**Ag58B (A)**	CR2106 (B)	Alkyl	ALA170 (A)

**Ag58B (A)**	CR2106 (B)	Alkyl	LEU41 (A)
**Ag58B (A)**	CR2106 (B)	Conventional Hydrogen Bond	ASN222 (A)
**Ag58B (A)**	CR2106 (B)	Pi-Pi T-shaped	TRP263 (A)
**Ag58B (A)**	CR2106 (B)	Conventional Hydrogen Bond	ARG42 (A)
**Ag58B (A)**	CR2106 (B)	Alkyl	LEU228 (A)

Interaction profile generated in Discovery Studio

Figure 1. Comparative physicochemical and structural profiles of B1CTcu5-derived peptides. Left: Helical-wheel projections assuming α-helical conformations, used here to illustrate the potential amphipathic distribution of residues. The presence of Trp in W-B1CTcu5 and CR2106 enhances hydrophobic face density. Center: Linear residue alignment depicting physicochemical class distribution. Color coding denotes residue type: basic (blue), aromatic (green), polar (light green), cysteine (yellow), aliphatic (gray). W-B1CTcu5 contains an *N*-terminal Trp and exhibits increased hydrophobic continuity and amphipathic polarity as compared to the parental scaffold B1CTcu5. CR2111 preserves the hydrophobic–hydrophilic balance with minimal structural divergence. Right: Theoretical net charge profiles as a function of pH, with all peptides maintaining positive charge at physiological pH, indicating a propensity for membrane interactions.

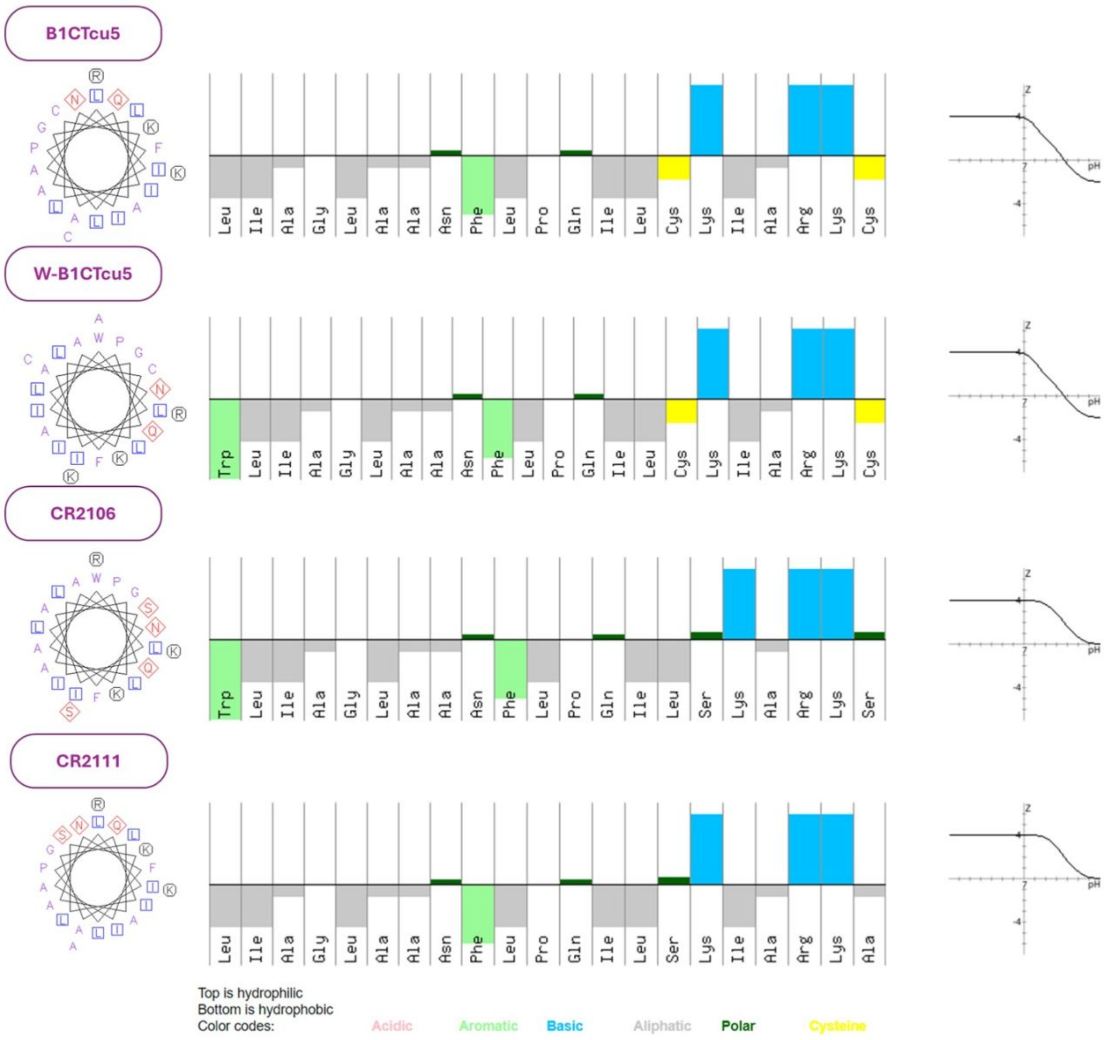

Figure S2. Ramachandran plots of all four peptides following 100 ns of molecular dynamics simulation. Each plot displays the distribution of backbone dihedral angles (φ and ψ) for B1CTcu5, CR2106, W-B1CTcu5, and CR2111. Blue dots represent the (φ, ψ) angles sampled throughout the trajectory. W-B1CTcu5 and CR2111 display clustering within the α-helical region, indicating stable secondary structure. In contrast, CR2106 shows broader dispersion of dihedral angles, suggesting conformational disorder and reduced structural persistence. These trends align with RMSD/RMSF data and support differential dynamic behavior influencing peptide bioactivity.

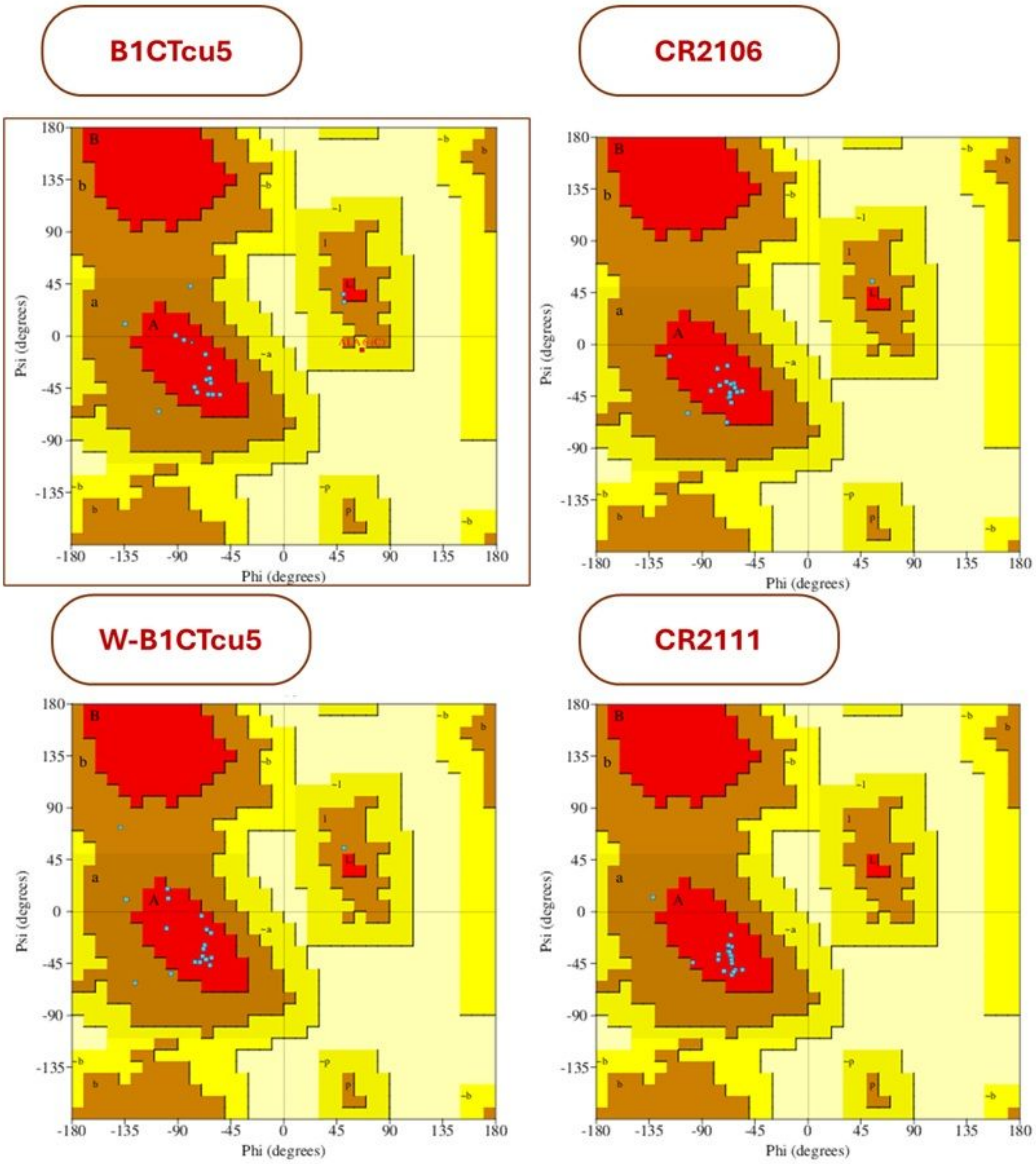

Figure S3–S6. Final peptide conformations at the end of the 100 ns molecular dynamics simulations in a mycolic acid bilayer. Snapshots illustrate the structural conformation and spatial positioning of each peptide after 100 ns of atomistic simulation in a mycolic acid membrane environment. Peptides are rendered as cartoons with distinct colors and shown embedded within the transparent lipid matrix:

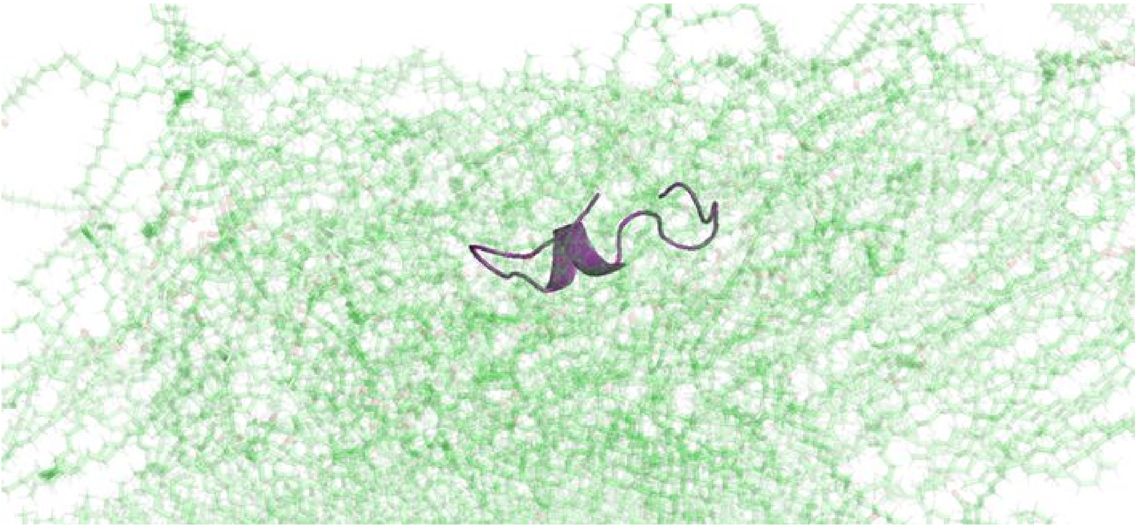

Figure S2. Final conformation of B1CTcu5, which retains partial folding but shows increased flexibility at the N-terminus.

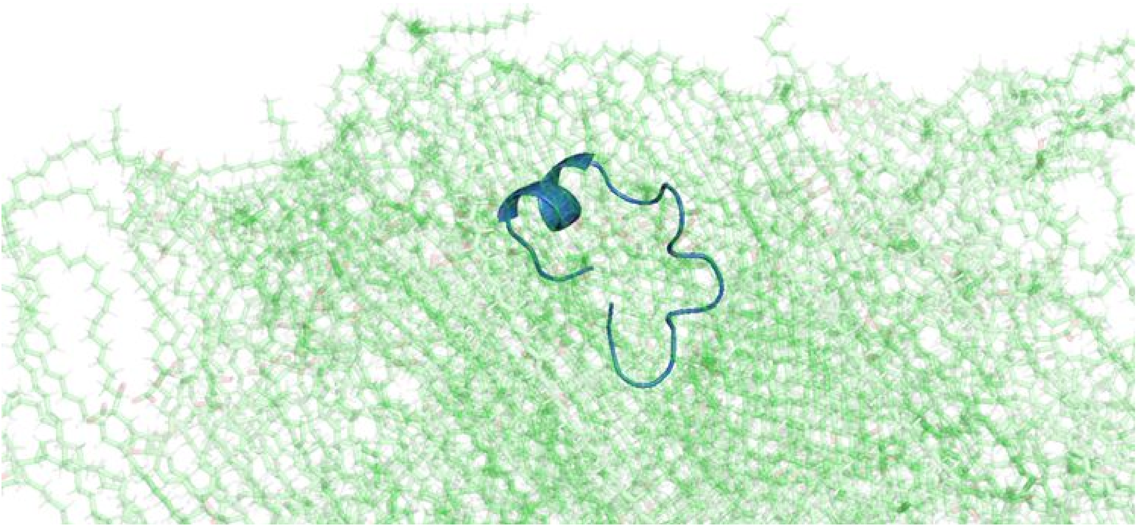

Figure S3. Final conformation of W-B1CTcu5, which maintains a compact α-helical structure centrally located at the membrane interface.

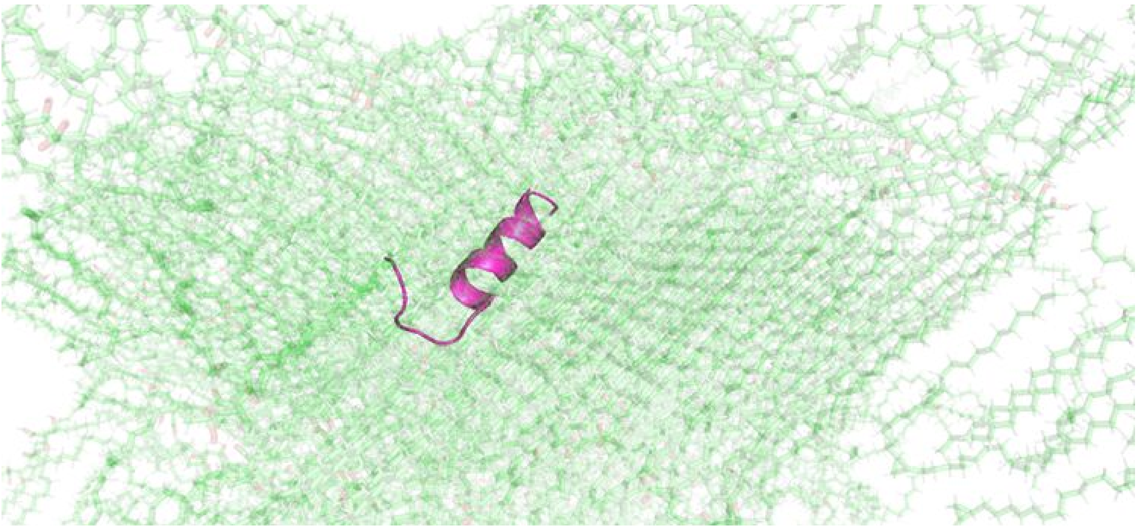

Figure S4. Final conformation of CR2106, showing deep insertion and notable structural unfolding, consistent with its dynamic instability.

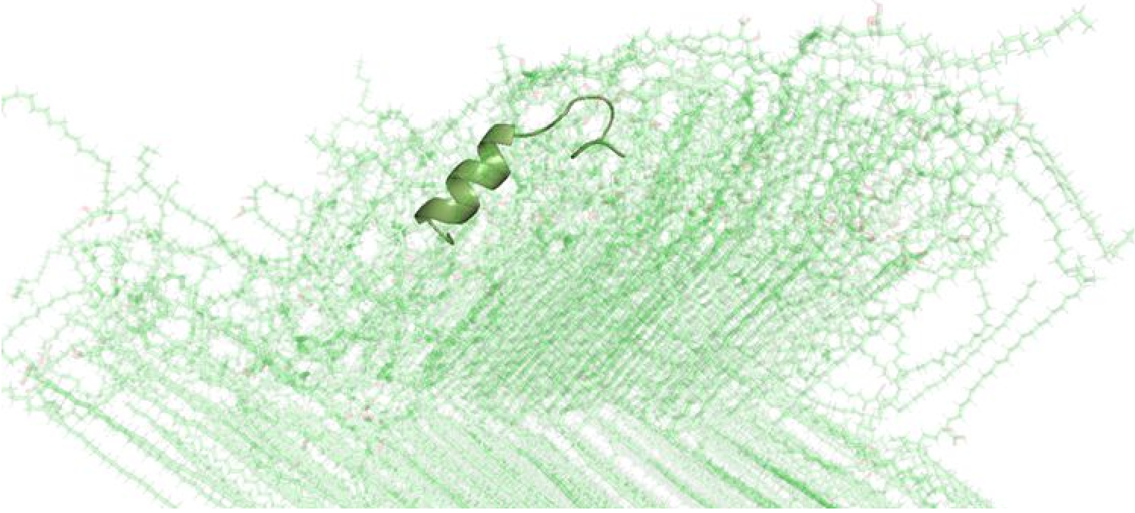

Figure S5. Final conformation of CR2111, exhibiting a stabilized central α-helix with moderate peripheral flexibility.

These structural end states visually support the RMSD and RMSF results discussed in the main text (Figure 5), highlighting the distinct folding and membrane-association behavior of each AMP.
